# Fused 1,5-Naphthyridines: Synthetic Tools and Applications

**DOI:** 10.3390/molecules25153508

**Published:** 2020-07-31

**Authors:** Carme Masdeu, Maria Fuertes, Endika Martin-Encinas, Asier Selas, Gloria Rubiales, Francisco Palacios, Concepcion Alonso

**Affiliations:** Departamento de Química Orgánica I, Facultad de Farmacia, Universidad del País Vasco/Euskal Herriko, Unibertsitatea (UPV/EHU), Paseo de la Universidad 7, 01006 Vitoria-Gasteiz, Spain; carmen.masdeu@ehu.eus (C.M.); maria.fuertes@ehu.eus (M.F.); endika_690@hotmail.com (E.M.-E.); asier.selas@ehu.eus (A.S.); gloria.rubiales@ehu.eus (G.R.)

**Keywords:** fused 1,5-naphthyridines, heterocycle synthesis, biological activity, metal complexes

## Abstract

Heterocyclic nitrogen compounds, including fused 1,5-naphthyridines, have versatile applications in the fields of synthetic organic chemistry and play an important role in the field of medicinal chemistry, as many of them have a wide range of biological activities. In this review, a wide range of synthetic protocols for the construction of this scaffold are presented. For example, Friedländer, Skraup, Semmlere-Wolff, and hetero-Diels-Alder, among others, are well known classical synthetic protocols used for the construction of the main 1,5-naphthyridine scaffold. These syntheses are classified according to the nature of the cycle fused to the 1,5-naphthyridine ring: carbocycles, nitrogen heterocycles, oxygen heterocycles, and sulphur heterocycles. In addition, taking into account the aforementioned versatility of these heterocycles, their reactivity is presented as well as their use as a ligand for metal complexes formation. Finally, those fused 1,5-naphthyridines that present biological activity and optical applications, among others, are indicated.

## 1. Introduction

Several reviews have appeared in the area of naphthyridines [1,2,3,4] including some references to fused 1,5-naphthyridines. Among them, in 2005, Ivanov et al. [5] published a generic review on benzo[*b*]naphthyridines. However, there are no previous reviews that specifically address the chemistry and application of fused 1,5-naphthyridines. For these reasons, to avoid overlapping with previous contributions, our wish in this review is coverage since 2003.

**Figure 1:**
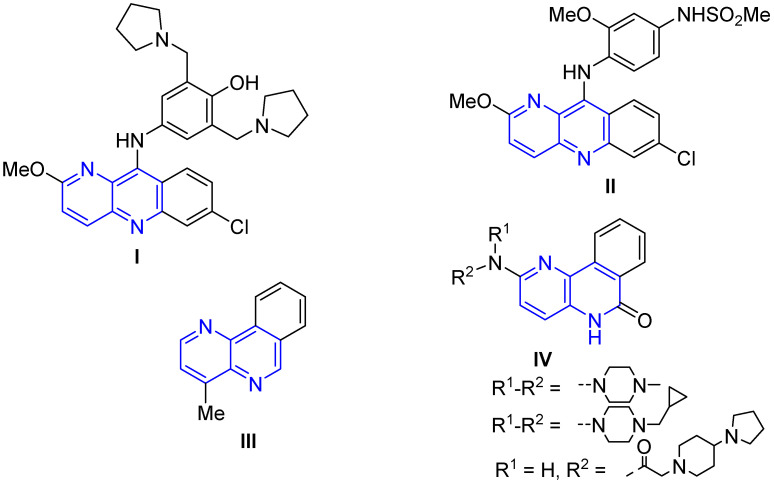
Fused naphthyridines with biological applications.

Heterocyclic compounds, such as fused 1,5-naphthyridines, are significantly important in the field of medicinal chemistry, because many of them present a wide variety of biological activities. For example, it was reported that pyronaridine **I** (Figure 1) has a high activity against *Plasmodium falciparum* and *Plasmodium vivax* [6,7]. Benzo[*b*][1,5]naphthyridine **II** (Figure 1) presented noticeable cytotoxicity against human HL-60 and HeLa cells grown in culture and topoisomerase II inhibition [8]. Moreover, through chemotherapy of solid-tumor-bearing mice, this compound **II** resulted as potent as amsacrine (m-AMSA) but less toxic towards the host. In 1994, Sliwa et al. showed that benzo[*b*]1,5-naphthyridine **III** (Figure 1) presented higher activity against Gram-positive than Gram-negative strains [9]. Years later, 5*H*-benzo[*c*][1,5]naphthyridin-6-ones **IV** (Figure 1) showed poly ADP ribose polymerase (PARP)-1 inhibition and protective effects in rat models of stroke and heart ischemia [10].

Regarding the organization of this review, first of all, the synthesis of 1,5-naphthyridines fused with carbocycles will be addressed, followed by the synthesis of 1,5-naphthyridines fused with nitrogen heterocycles, fused with oxygen heterocycles, and fused with thieno heterocycles. Afterwards, the reactivity of fused 1,5-naphthyridines is classified by *N*-alkylation, electrophilic substitution reactions (S_E_Ar), nucleophilic substitution reactions (S_N_Ar), oxidations, reductions, side chain modifications, and metal complex formation. Finally, some properties and applications of these heterocycles studied during this period are reviewed.

## 2. Synthesis of Fused 1,5-Naphthyridines

### 2.1. Synthesis of 1,5-Naphthyridines Fused with Carbocycles

**Figure 2:**
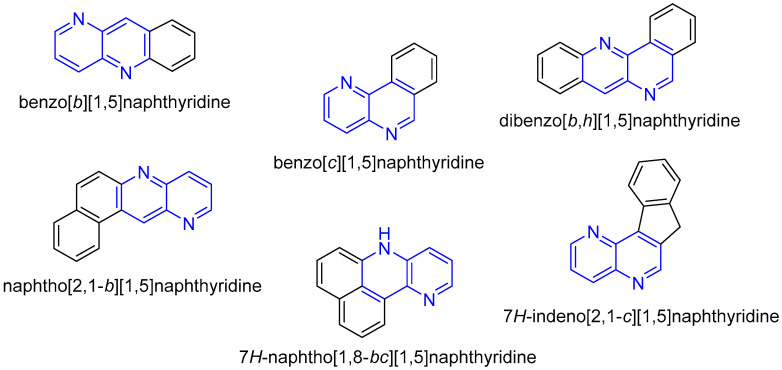
Examples of fused 1,5-naphthyridines with carbocycles.

In this section, general methods of synthesis for 1,5-naphthyridines fused with benzene, naphthalene, and indene rings (Figure 2) are analyzed.

**Scheme 1:**
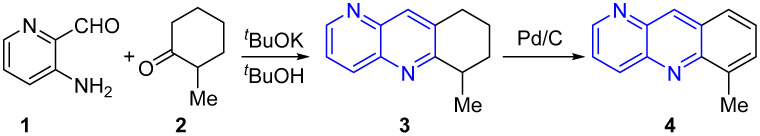
Preparation of benzo[*b*][1,5]naphthyridines by Friedländer reaction.

The most commonly used synthetic routes for the synthesis of benzo[*b*][1,5]naphthyridines are based on the Friedländer reaction and on the Skraup synthesis using 3-aminopyridine or 3-aminoquinoline derivatives and carbonyl compounds. There are numerous examples collected in the excellent reviews published previously [1,3,4,5] describing these approaches. As an example, the reaction of 3-aminopicolinaldehyde **1** with 2-methylcyclohexanone **2** (Scheme 1) in *^t^*BuOH in the presence of *^t^*BuOK followed by dehydrogenation of the 6,7,8,9-tetrahydrobenzo[*b*][1,5]naphthyridine **3**, formed at reflux with Pd/C in Ph_2_O, afforded benzo[*b*][1,5]naphthyridine **4** [4].

**Scheme 2:**
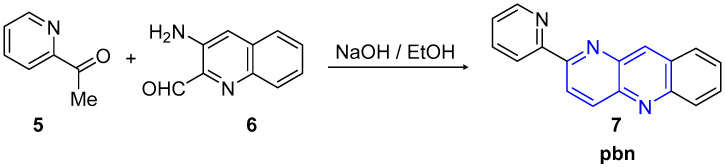
A modified Friedländer reaction for the preparation of pbn ligand.

Similarly, the ligand 2-(pyridin-2-yl)benzo[*b*][1,5]naphthyridine **7** (pbn) was prepared by a modified Friedländer reaction of 3-aminoquinaldehyde **6** with 2-acetylpyridine **5** (Scheme 2) in ethanol in the presence of NaOH [11,12]. As will be seen later, this compound has been used as a ligand in the preparation of Ru, Rh, and Pd complexes to study its electrochemical behavior.

**Scheme 3:**
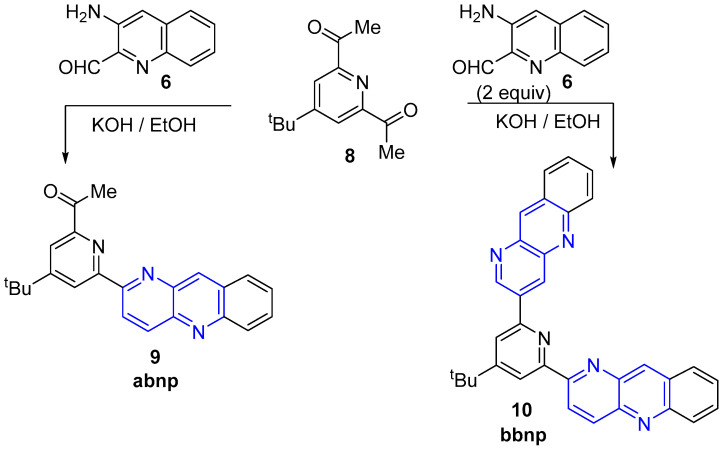
Friedländer reaction for the synthesis of benzo[*b*][1,5]naphthyridine derivatives **9** and **10**.

An analogous reaction was used for the preparation of 2-acetyl-6-benzo[*b*][1,5]naphthyridin-2-yl-4*-tert*-butylpyridine **9** (abnp, Scheme 3) and 2,6-bis(benzo[*b*][1,5]naphthyridin-2-yl)-4-*tert*-butylpyridine **10** (bbnp, Scheme 3) [13]. In this case, the Friedländer reaction between equimolar amounts of quinoline **6** and 2,6-diacetyl-4-*tert*-butylpyridine **8** gave derivative **9** (abnp)**,** while compound **10** (bbnp) was obtained by condensing 2,6-diacetyl-4-*tert*-butylpyridine **8** with two equivalents of 3-aminoquinaldehyde **6**.

**Scheme 4:**
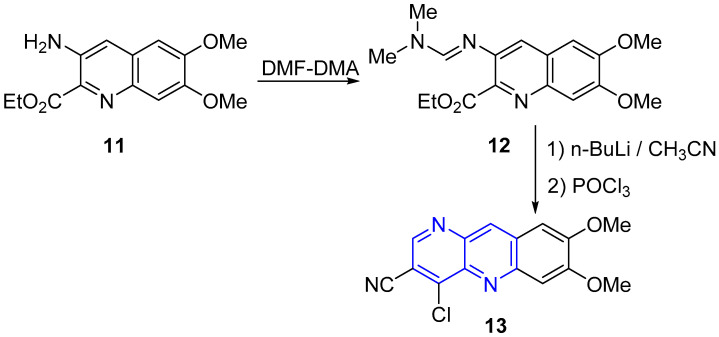
Formation of a cyano benzo[*b*][1,5]naphthyridine derivative in two steps.

In a similar way, 3-aminoquinoline-2-carboxylate **11** (Scheme 4) was transformed into benzo[*b*][1,5]naphthyridine **13** in two steps [14]. The reaction of quinoline **11** with the DMF acetal gave amidine **12**, whose reaction with the acetonitrile anion followed by chlorination with phosphorus oxychloride provided derivative **13**.

**Scheme 5:**
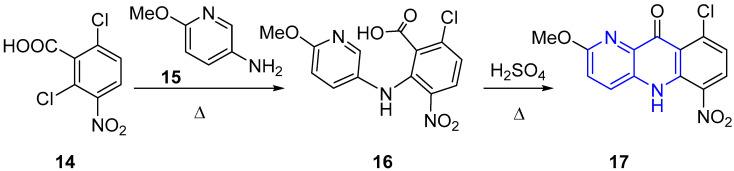
Modified Skraup synthesis of 5,10-dihydrobenzo[*b*][1,5]naphthyridin-10-one.

A modified Skraup synthesis allows the preparation of 5,10-dihydrobenzo[*b*][1,5]naphthyridin-10-one **17** (Scheme 5) by heating at 75 °C the 2,6-dichloro-3-nitrobenzoic acid **14** in 6-methoxy-3-pyridinamine **15**. Thus, the 6-chloro-2-[(6-methoxy-3-pyridyl)amino]-3-nitrobenzoic acid **16** was obtained. Heating of **16** at 100 °C in H_2_SO_4_ leads to the 9-chloro-2-methoxy-6-nitro-5,10-dihydrobenzo[*b*][1,5]naphthyridin-10-one **17** [15].

**Scheme 6:**
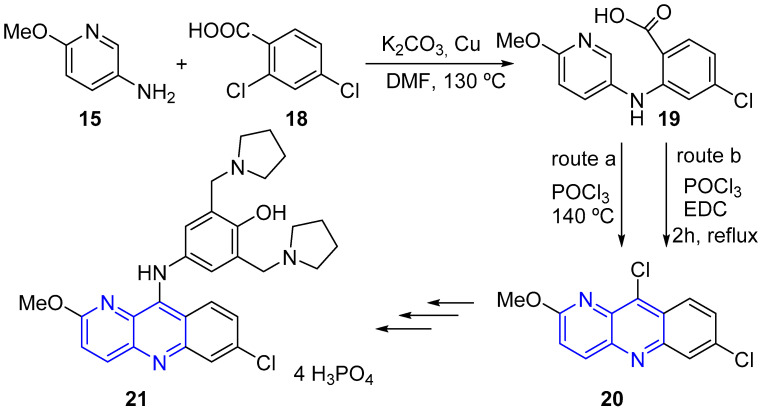
Skraup synthesis of 9-chloroacridine derivative.

In a similar way, when anthranilic acid derivative **19** was stirred with POCl_3_ at 140 °C (Scheme 6, route a), the 9-chloroacridine derivative **20** was obtained [16]. A new approach to the preparation of anthranilic acid **19** is a nucleophilic substitution reaction by heating 2,4-dichlorobenzoic acid **18** with 5-amino-2-methoxypyridine **15** in the presence of Cu (Scheme 6).

The process has been applied to the industrial preparation of pyronaridine tetraphosphate **21** (Scheme 6), a well-known antimalarial drug, by introducing several modifications [17]. The cyclization of compound **19** to derivative **20** is the most important stage in the synthesis of pyronaridine tetraphosphate **21**. However, purification of compound **20** was difficult because of its poor solubility in common solvents. In order to reduce undesired impurities produced during reaction, ethylene dichloride (EDC) was used as solvent and short reaction times were applied to the process (Scheme 6, route b). Following this improved manufacturing process, 9-chloroacridine **20** was synthesized in a high purity, increased yield and lower production cost.

**Scheme 7:**
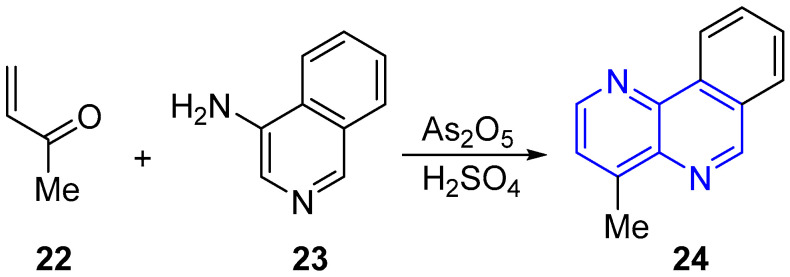
Michael addition of 4-aminoisoquinoline to methyl vinyl ketone for the formation of **24**.

Synthetic routes based on Skraup synthesis are also convenient approaches to obtain benzo[*c*][1,5]naphthyridines. A modified Skraup method, namely, Michael addition of 4-aminoisoquinoline **23** (Scheme 7) with methyl vinyl ketone **22** in the presence of As_2_O_5_ and concentrated sulfuric acid, was used to prepare 4-methylbenzo[*c*][1,5]naphthyridine **24** [4].

**Scheme 8:**
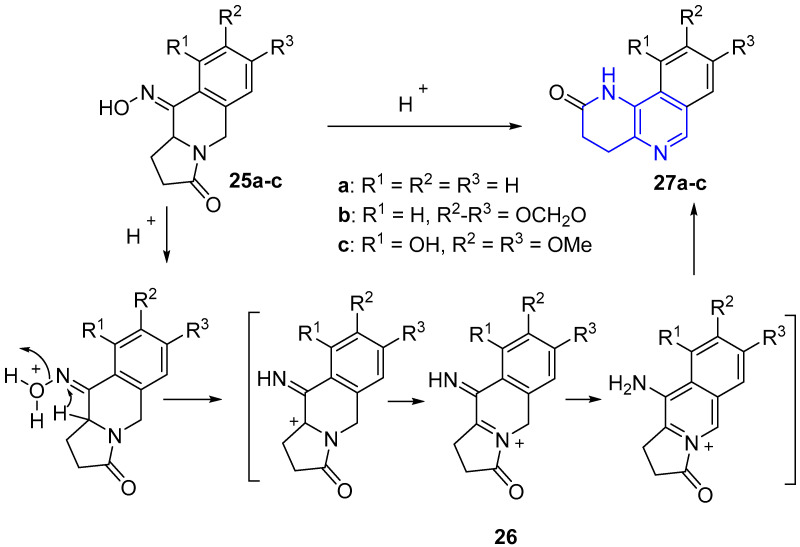
Formation of 3,4-dihydrobenzo[*c*][1,5]naphthyridin-2(1*H*)-ones by a Semmlere–Wolff transposition of oximes.

On the other hand, the treatment of oximes **25a**–**c** with acid (Scheme 8), allows the formation of 3,4-dihydrobenzo[*c*][1,5]naphthyridin-2(1*H*)-ones **27a**–**c** [18]. The formation of derivatives **27a**–**c** takes place through a Semmlere–Wolff transposition (Scheme 8) with good yields. It is important to note that the mechanism leading to derivatives **27a**–**c** involves the formation of a stabilized *N*-acyliminium salt **26** followed by the opening of the lactam ring.

**Scheme 9:**
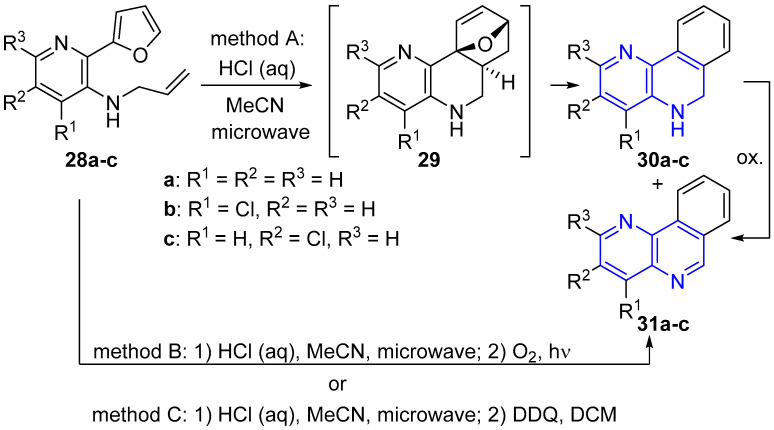
Synthesis of benzo[*c*][1,5]naphthyridines through a [4+2] cycloaddition reaction.

The preparation of benzo[*c*][1,5]naphthyridines can be also performed by [4+2] cycloaddition reaction. Thus, the microwave-mediated intramolecular Diels–Alder (DA) reaction [19] of *o*-furyl(allylamino)pyridines **28a**–**c**, in the presence of a catalytic amount of acid, gave 5,6-dihydrobenzo[*c*][1,5]naphthyridines **30a**–**c** (Scheme 9, method A). The initially formed DA-adduct **29** spontaneously underwent ring opening and subsequent aromatization to afford the 5,6-dihydrobenzo[*c*][1,5]naphthyridines **30a**–**c**. Electron-withdrawing substituents, especially R^1^ = Cl, seem to stabilize the 5,6-dihydrobenzo[*c*][1,5]naphthyridines **30**. Some of these dihydro compounds **30** were oxidized, with high yields, to the aromatic compounds **31** during workup and purification. When the reaction mixture was bubbled through air in the presence of UV light (Scheme 9, method B) or stirred with 2,3-dichloro-5,6-dicyano-*p*-benzoquinone (DDQ, 1.2 equivalent) at room temperature (Scheme 9, method C) aromatic benzo[*c*][1,5]naphthyridine compounds **31a**–**c** were obtained.

**Scheme 10:**
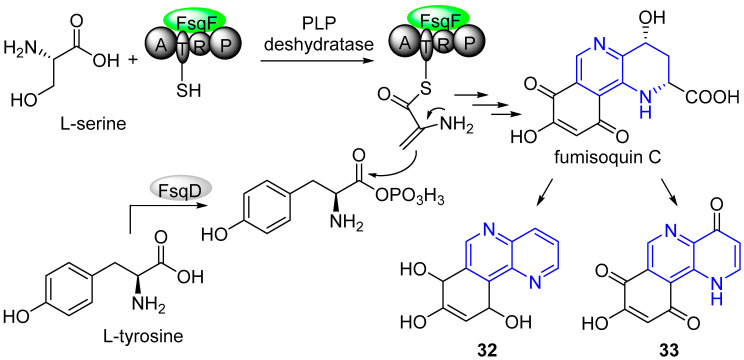
Microbial biosynthesis of benzo[*c*][1,5]naphthyridine alkaloids.

Through metabolomics, several benzo[*c*][1,5]naphthyridine alkaloids, known as fumisoquins, have been prepared. For this purpose, groups of microbial biosynthetic gene clusters (BGC) in the human pathogen *Aspergillus fumigatus* called fsq have been used [20]. The authors demonstrated that fsqF, which lacks a canonical condensation domain, is necessary for the formation of carbon–carbon bonds between the amino acids l-serine and l-tyrosine in the fumisoquin biosynthetic pathway. The fsqD appears to activate tyrosine for subsequent condensation with serine-derived dehydroalanine (Scheme 10), which is the first example of a new strategy for the formation of carbon–carbon bonds in fungi. Optimization of the extraction conditions and reverse phase fractionation followed by two-dimensional NMR spectroscopy and HRMS analysis allowed the identification of fumisoquin C (Scheme 10) as the deep purple metabolite. While standing or during chromatography, fumisoquin C decomposes into **32** and **33** (Scheme 10) which are more stable and were isolated.

**Scheme 11:**
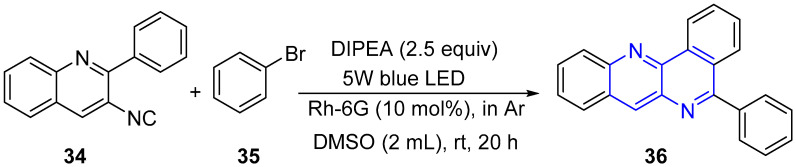
Rhodamine 6G synthesis of 5-phenyldibenzo[*b*,*h*][1,5]naphthyridine derivative.

A visible-light-catalyzed synthesis of 5-phenyldibenzo[*b,h*][1,5]naphthyridine **36** from 3-isocyano-2-phenylquinoline **34** and bromobenzene **35** at room temperature has been discovered [21]. This metal-free cross-coupling reaction offers rapid and sustainable access to a series of structurally complex dibenzo[*b,h*][1,5]naphthyridines **36** (Scheme 11). The usage of inexpensive Rhodamine 6G (Rh-6G) as the catalyst with easy operation makes this protocol very practical. A plausible mechanism was proposed through a radical anion [Rh-6G•-] formed by the use of the visible-light irradiation, that triggers a single electron transfer to bromobenzene producing the transient [Ph-Br•-] radical anion and regenerating Rh-6G completing the catalytic cycle.

**Scheme 12:**
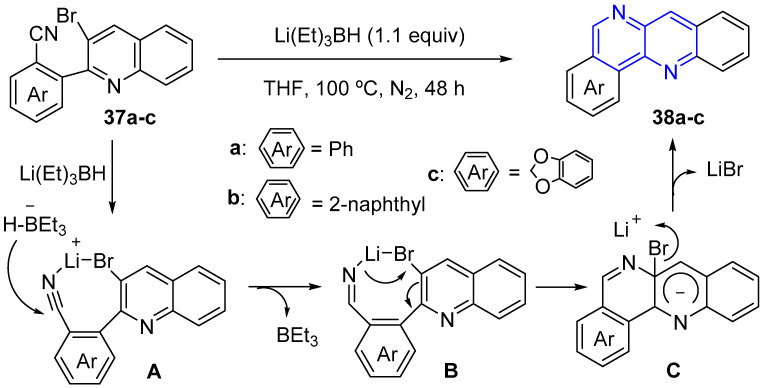
Synthesis of benzo[*b*][1,5]naphthyridines by reduction of biaryl bromonitriles.

A novel procedure for hydride-induced anionic cyclization has been developed. It includes the reduction of a biaryl bromonitrile **37** with a nucleophilic aromatic substitution (S_N_Ar). Dibenzo[*b,h*][1,5]naphthyridine **38a** and benzo[*b*]naphtho[2,3-*h*][1,5]naphthyridine **38b** were so obtained in moderate-to-good yield with good substrate tolerance (Scheme 12). In addition, the analogue of trisphaeridine **38c** could be also obtained in moderate yield [22]. This method involves a concise transition-metal-free process, and it was applied to synthesize natural alkaloids. A tentative reaction pathway can be proposed as below (Scheme 12). The reaction is likely to be initiated by the chelating of compound **37** to lithium (complex **A**). Subsequent addition of hydride to nitrile provides the iminyl lithium complex (complex **B**). A subsequent nucleophilic aromatic substitution then takes place to generate the anionic intermediate **C**. Elimination of halide affords desired products **38** and precipitate LiBr.

**Scheme 13:**
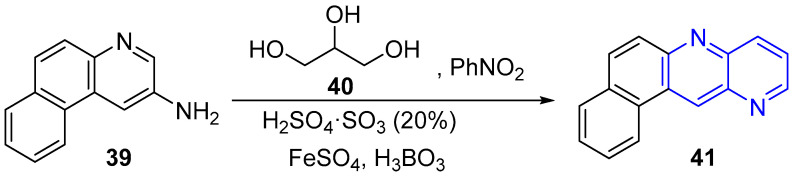
Skraup synthesis of a naphtho[2,1-*b*][1,5]naphthyridine.

Naphtho[2,1-*b*][1,5]naphthyridines can also be prepared by the Skraup synthesis. Thus, annulated naphthonaphthyridine **41** (Scheme 13) was synthesized starting from 2-aminobenzo[*f*]quinoline **39** [4,5]. This reaction was carried out with glycerol **40** in the presence of an oxidant (nitrobenzenesulfonic acid) formed in situ upon the reaction of nitrobenzene with *oleum*.

**Scheme 14:**
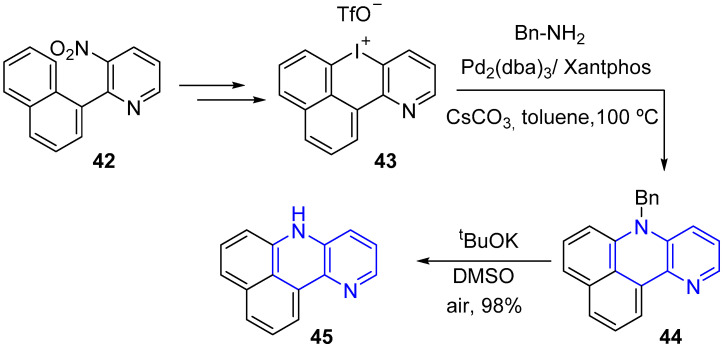
Formation of naphtho[1,8-*bc*][1,5]naphthyridines using nitropyridylnaphthalene.

Nitropyridylnaphthalene **42** is the central intermediate for the synthesis of naphtho[1,8-*bc*][1,5]naphthyridine derivatives [23]. Transformation of the nitro group to iodo followed by oxidation and cyclization results in an iodonium salt **43** (Scheme 14). Using the successful protocol for the transformation of annulated iodolium salts to pyrroles**,** Buchwald-Hartwig (Palladium-catalyzed arylamination) with benzylamine (Bn-NH_2_) was achieved with **43** to give compound **44**. The unsubstituted 7*H*-naphtho[1,8-*bc*][1,5]naphthyridine **45** was obtained in nearly quantitative yield via base-induced debenzylation of **44** (Scheme 14).

The first report for the synthesis of substituted 7*H*-indeno[2,1-*c*][1,5]naphthyridine derivatives was carried out by Palacios and colleagues [24,25,26].

**Scheme 15:**
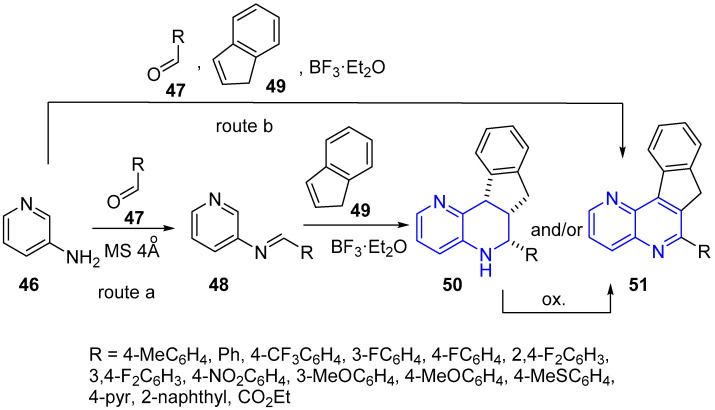
Povarov reaction for the preparation of substituted 7*H*-indeno[2,1-*c*][1,5]naphthyridines.

The process consists of a Povarov-type [4+2]-cycloaddition reaction, by both step-by-step and by multicomponent strategies (MCRs). First, the hetero-Diels-Alder reaction between indene **49** (Scheme 15, route a) and *N*-(3-pyridyl) aldimines **48**, prepared in situ by reaction of 3-pyridylamine **46** and aldehydes **47**, in the presence of two equivalents of BF_3_·Et_2_O in refluxing chloroform were performed. Afterwards, the corresponding tetracyclic *endo*-1,2,3,4-tetrahydro[1,5]naphthyridines **50** were selectively obtained with good yields (route a, Scheme 15) in a regio- and stereospecific way. Alternatively, three-component synthetic protocol was carried out (Scheme 15, route b) by reacting commercially available 3-pyridylamine **46**, aromatic aldehydes **47** and indene **49** in the presence of 2 equivalents of BF_3_·Et_2_O in refluxing chloroform to afford also the corresponding *endo*-1,2,3,4-tetrahydronaphthyridines **50** with good yields. The presence of electron-donating groups (OMe and SMe) seems to favor the direct formation of indeno[1,5]naphthyridines **51**. Formation of these derivatives could be reasoned by a [4+2]-cycloaddition reaction between aldimines **48** and indene **49** and, subsequent, dehydrogenation of tetrahydroindeno[1,5]naphthyridines **50**. The scope of this strategy is very wide, given that compounds **50** and **51** substituted not only with a pyridine group (R = 4 pyr) but also with a wide range of *ortho*, *meta,* and *para* aromatic substrates containing electron-releasing and -withdrawing groups, including fluorine substituents can be prepared. 

### 2.2. Synthesis of 1,5-Naphthyridines Fused with Nitrogen Heterocycles

Nitrogen atoms are ubiquitous in biologically significant secondary metabolites (alkaloids, cytokinins), in biomacromolecules (proteins, peptides, DNA, RNA) as well as in synthetic organic substances, and often belong to atomic centers of importance for intra- and intermolecular interactions. Therefore, the addition of nitrogen atoms to a structure can contribute to the discovery and development of new therapeutic agents for the treatment of different diseases, which represents one of the most important objectives in medicinal chemistry.

**Figure 3:**
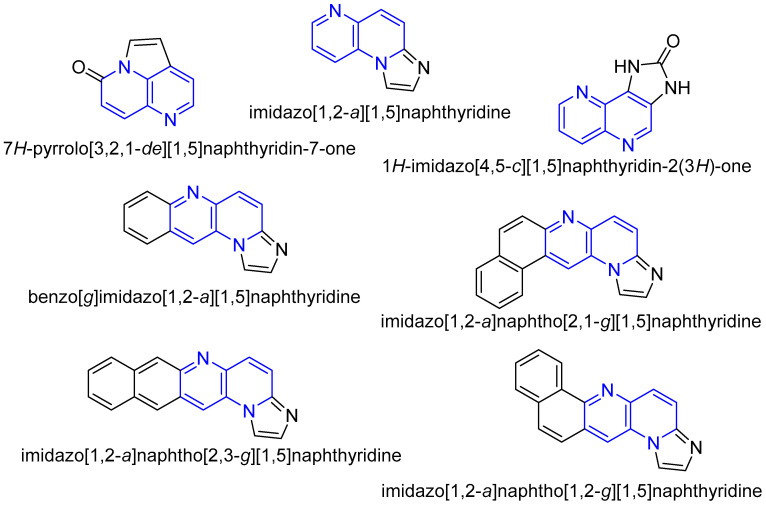
Examples of fused 1,5-naphthyridines with five-membered nitrogen heterocycles.

This section, firstly, will analyze the general methods of synthesis of 1,5-naphthyridine fused with five-membered nitrogen heterocycles (Figure 3).

**Scheme 16:**
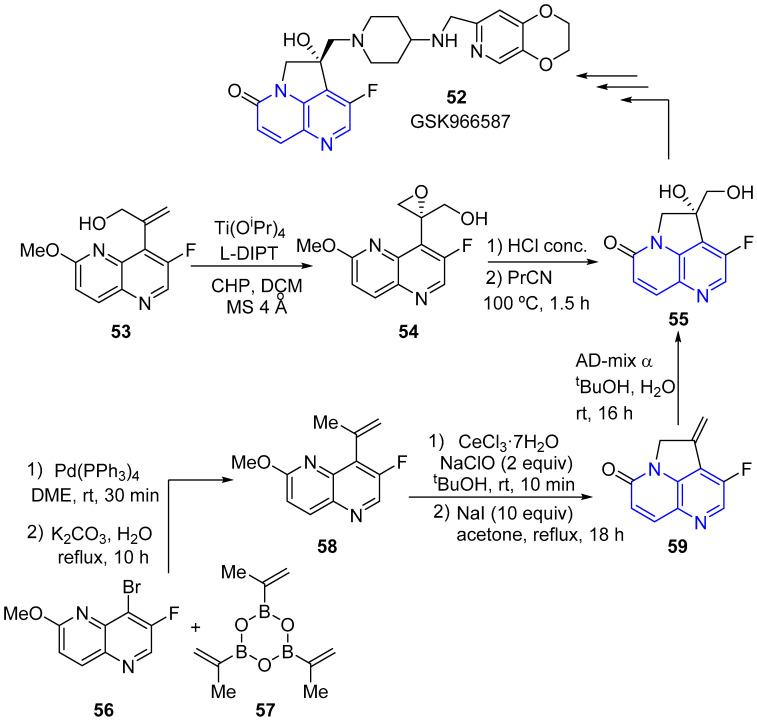
Enantioselective total synthesis of the potent antibiotic GSK966587.

An efficient enantioselective total synthesis of the potent antibiotic GSK966587 **52** (Scheme 16) was accomplished [27]. After the synthesis of the corresponding fused 1,5-naphthyridine and several structural modifications, the desired allylic alcohol **53** was obtained. A Sharpless asymmetric epoxidation of allylic alcohol **53** gives epoxy alcohol **54**. When the unpurified epoxy alcohol **54** was treated with concentrated HCl and after aqueous workup, the solution was heated in butyronitrile to 100 °C for 1.5 h, causing cyclization and demethylation in one pot. Cooling to room temperature allowed the crystallization of tricyclic diol **55** from allylic alcohol **53** (Scheme 16). The preparation of the fully elaborated side chain and various final transformations gave GSK966587 as a precipitate directly from the reaction mixture.

The synthesis of 4,5-dihydro-6λ^4^pyrrolo[3,2,1-*ij*][1,5]naphthyridine derivatives, including a new synthesis of **52** GSK966587, has also been reported [28]. In this case, a Suzuki cross-coupling of 8-bromo-7-fluoro-2-methoxy[1,5]naphthyridine **56** (Scheme 16) with pyridine-tris (1-methylethenyl) boroxin **57** gives **58** (Scheme 16). Then, chlorination with cerium (III) chloride heptahydrate gave a chloride whose cyclisation using NaI in acetone during 18 h of reflux afforded 3-fluoro-4-methylene-4*H*-pyrrolo[3,2,1-*de*][1,5]naphthyridin-7(5*H*)-one **59**. Then, subsequent dihydroxylation yielded the previously described diol **55** (Scheme 16).

**Scheme 17:**
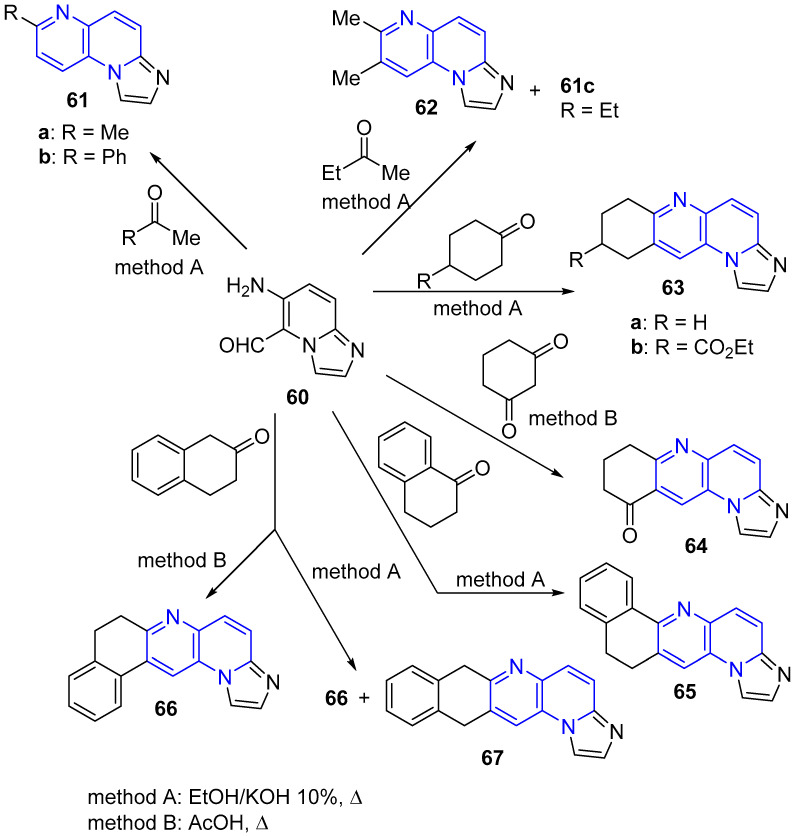
Modified Friedländer reactions for the synthesis of imidazo[1,2-*a*][1,5]naphthyridines.

The preparation of imidazo[1,2-*a*][1,5]naphthyridinic systems was achieved by a modified Friedländer’s reaction [29]. In this case, from 6-aminoimidazo[1,2-*a*]pyridine-5-carbaldehyde **60** and ketones, such as acetone, butanone, acetophenone, cyclohexanone, ethyl 4-oxocyclohexane carboxylate, 1,3-cyclohexandione, α- or β-tetralone, the corresponding compounds were obtained using EtOH/KOH 10% (Scheme 17, method A) or AcOH (Scheme 17, method B). The protocol is effective for the synthesis of imidazo[1,2-*a*][1,5]naphthyridines **61a**–**c** and **62** and also for the synthesis of benzo[*g*]imidazo[1,2-*a*][1,5]naphthyridines **63a** and **63b**, from cyclohexanone and ethyl 4-oxocyclohexane**,** respectively (Scheme 17, method A). In contrast, compound **64** could be prepared by reaction of **60** with 1,3-cyclohexadione in an acetic acidic medium (Scheme 17, method B). Imidazo[1,2-*a*]naphtho[1,5]naphthyridines **65**–**67** were prepared from α- or β-tetralone, respectively. Under alkaline conditions (Scheme 17, method A) α-tetralone gave the single dihydro compound **65**, while β-tetralone yielded the compounds **66** and **67**. It is interesting to note that the use of acidic conditions (Scheme 17, method B) afforded only compound **66**.

**Scheme 18:**
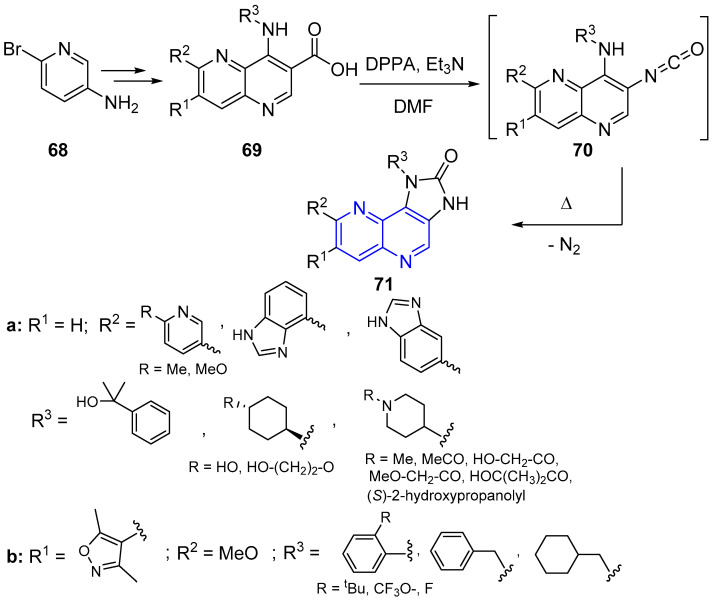
Preparation of tricyclic imidazo[4,5-*c*][1,5]naphthyridine derivatives.

The tricyclic system of imidazo[4,5-*c*][1,5]naphthyridine present in a series of compounds **71a** (R^1^ = H) and in the potent and selective PI3K/mTOR dual kinase inhibitor PF-04979064, was prepared [30] from **68** after several modifications to obtain **69** (Scheme 18). When **69** is treated with diphenylphosphoryl azide (DPPA) and Et_3_N, the initially formed nitrene intermediate gives isocyanate **70** which by Curtius rearrangement reacts with the amine group at 4-naphthyridine to undergo intramolecular cyclization producing the 1,3-dihydro-2*H*-imidazo[4,5-*c*][1,5]naphthyridin-2-ones **71** (Scheme 18). Following these strategies, tricyclic derivatives **71a** were designed with phenyl substituted cyclohexane and piperidine substituents. In a similar way, imidazo [4,5-*c*][1,5]naphthyridines **71b** (Scheme 18), that possess an isoxazole substituent (R^1^ = 3,5-dimethylisoxazol-4-yl), were synthesized from the corresponding previously prepared carboxylic acids **69b** [31].

**Figure 4:**
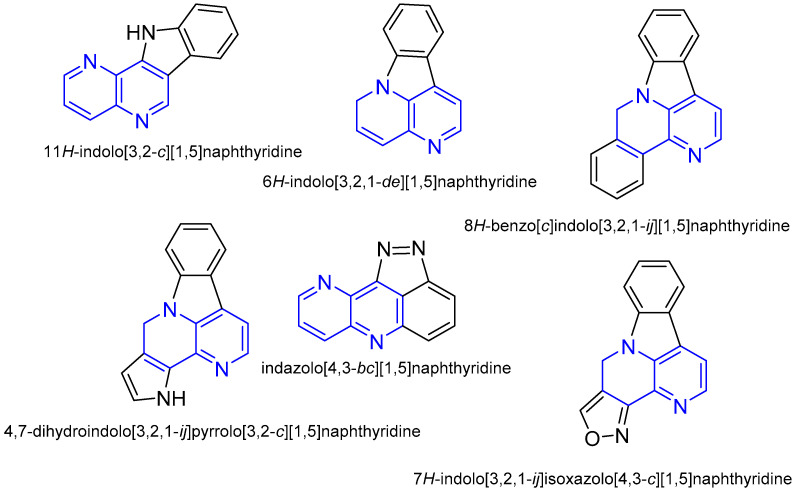
Examples of indolo and indazolo[1,5]naphthyridine derivatives.

Indole framework holds a very high affinity to multiple receptors and enzymes and, accordingly, it is considered a privileged structure in many active medicine compounds for human health and represents a promising scaffold for drug development (Figure 4) [32,33,34,35].

**Scheme 19:**
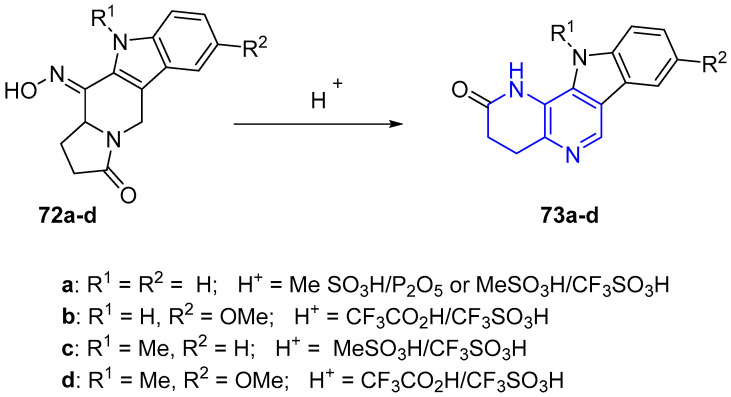
Preparation of indol 1,5-naphthyridines by a Semmier–Wolf transposition of oximes.

Indol systems fused to the 1,5-naphthyridine nucleus can be obtained by heating oximes in an acidic medium, through a Semmier-Wolf transposition, similar to the preparation of 3,4-dihydrobenzo[*c*][1,5]naphthyridin-2(1*H*)-ones **27** previously described (Scheme 8, *vide supra*) [18]. No general conditions were found for the transformation of oximes **72a**–**d** into the corresponding tetrahydro-2*H*-indolo[3,2*-c*][1,5]naphthyridin-2-ones **73a**–**d** (Scheme 19), and it was necessary to adjust the media according to the substituents present in the aromatic group. The advantage of using triflic acid mixtures in trifluoroacetic or methanesulfonic acid is the easy quenching of the reaction media when large amounts of oximes are employed.

**Scheme 20:**
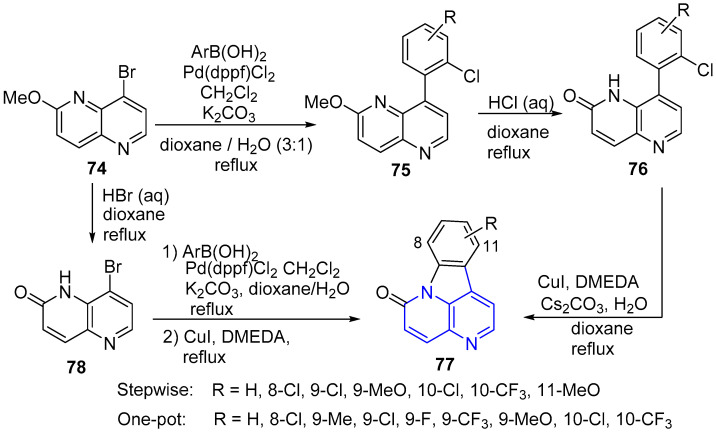
Syntheses of canthin-6-ones through a Suzuki-Miyaura coupling.

Two high-yielding and flexible syntheses of canthin-6-ones **77** (R = H, Scheme 20) were developed and optimized [36]. In the first one, through a stepwise approach, the desired 8-(2-chlorophenyl)-2-methoxy[1,5]naphthyridine derivatives **75** were prepared from 8-bromo-2-methoxynaphthyridine **74** and 2-chlorophenylboronic acids via a Suzuki–Miyaura coupling. Reflux of the corresponding 8-aryl-2-methoxynaphthyridines **75** with aqueous HCl in dioxane gave naphthyridones **76**. The C-N coupling using CuI (10 mol%) and *N,N’*-dimethylethylenediamine (DMEDA, 20 mol%) gave six new 6*H*-indolo[3,2,1-*de*][1,5]naphthyridin-6-ones **77** as well as canthin-6-one (R = H) in good overall yields.

The second one was a simple and useful one-pot protocol involving a sequential application of a Pd-catalyzed Suzuki-Miyaura coupling of naphthyridine **78** prepared from 8-bromo-2-methoxynaphthyridine **74** (Scheme 20), followed by a Cu-catalyzed amidation, of general use for synthetic chemists. Using this new one-pot protocol, nine 6*H*-indolo[3,2,1-*de*][1,5]naphthyridin-6-ones **77** with various substituents in the aromatic ring were quickly obtained in excellent yields (Scheme 20).

**Scheme 21:**
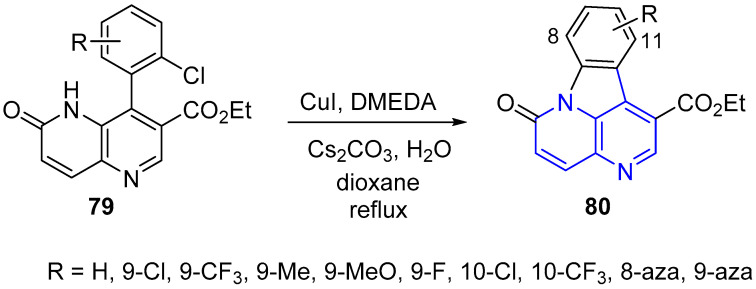
Copper-catalyzed Buchwald cyclization to ethyl canthin-6-one-1-carboxylates.

Via a copper-catalyzed Buchwald cyclization, ethyl canthin-6-one-1-carboxylate **80** (R = H, Scheme 21) and nine analogues were obtained from ethyl 4-(2-haloaryl)-6-oxo-5,6-dihydro [1,5]naphthyridine-3-carboxylates **79**. Thus, treatment of **79** with typical Buchwald conditions [CuI (5 mol%), DMEDA (10 mol%), Cs_2_CO_3_ (2 equivalents), water (2 equivalents)] in refluxing dioxane, gave the ethyl canthinone-1-carboxylates **80** [37]. A series of eight ethyl 6-oxo-6*H*-indolo[3,2,1-*de*] [1,5]naphthyridine-1-carboxylates **80** was prepared bearing various substituents in the aromatic ring, together with the 8-aza and 9-aza analogues (Scheme 21).

**Scheme 22:**
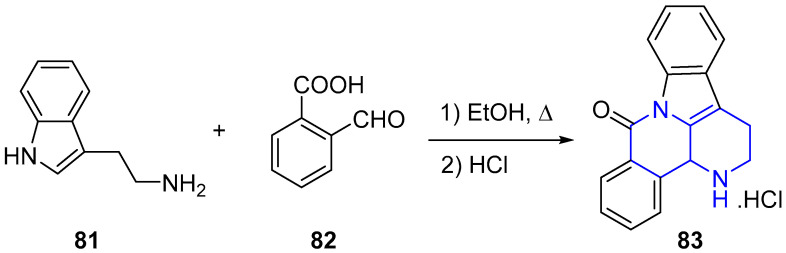
Formation of hexahydrobenzo[*h*]indolo[3,2,1-*de*][1,5]naphthyridine hydrochloride.

Tryptamine, tryptophan, and their derivatives are widely used in the synthesis of benzoindolo[3,2,1-*de*][1,5]naphthyridine derivatives [4]. Thus, refluxing tryptamine **81** with 2-formylbenzoic acid **82** in ethanol followed by the addition of concentrated hydrochloric acid afforded hexahydrobenzo[*h*]indolo[3,2,1-*de*][1,5]naphthyridine hydrochloride **83** (Scheme 22).

**Scheme 23:**
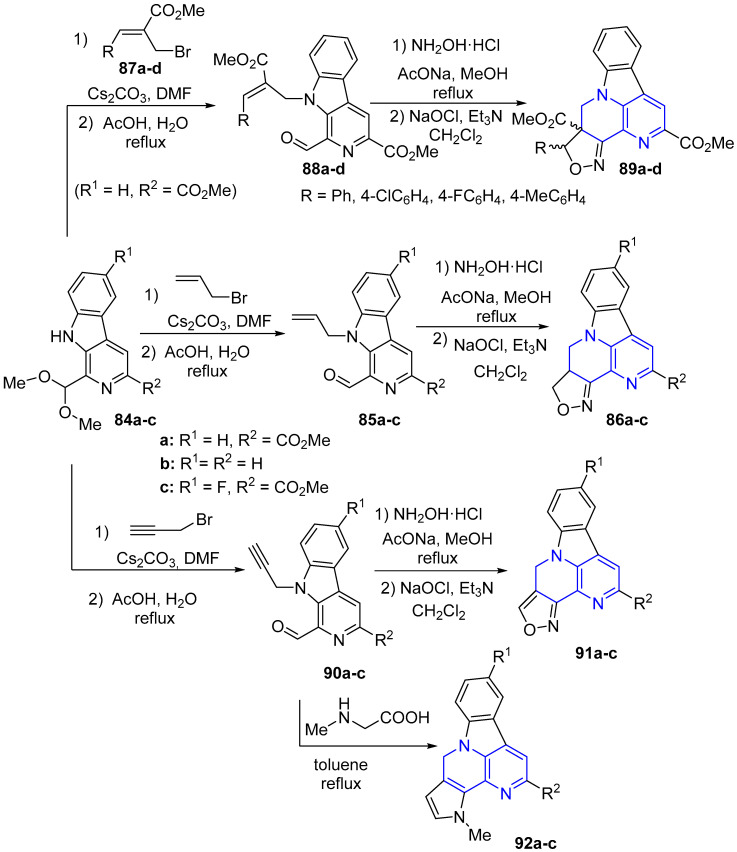
Synthesis of fused isoxazole and pyrrol[1,5]naphthyridine derivatives.

The synthesis of a library of β-carboline fused systems was carried out via intramolecular 1,3-dipolar cycloaddition reactions [38] starting from β-carboline protected aldehydes **84** (Scheme 23). Thus, protected aldehydes **84a**–**c** reacted with allyl bromide in the presence of Cs_2_CO_3_ as base in dry DMF, the acetal group was deprotected by heating in the presence of AcOH/water (2:3, *v*/*v*) at 120 °C and aldehydes **85a**–**c** were obtained. In the next step, the aldehydes **85a**–**c** reacted with NH_2_OH**·**HCl in the presence of NaOAc to furnish the substituted oximes, whose treatment with NaOCl in the presence of Et_3_N at room temperature for three days resulted in the formation of the desired 9a,10-dihydro-9*H*-indolo[3,2,1-*ij*]isoxazolo[4,3-*c*][1,5]naphthyridines **86a**–**c** (Scheme 23).

When the reaction of compound **84a** (R^1^ = H, R^2^ = CO_2_Me) with several substituted allyl bromides **87a**–**d** in the presence of Cs_2_CO_3_ in dry DMF is performed (Scheme 23) and the acetal group of obtained substituted alkenes **(***E*-isomer exclusively) is deprotected as described earlier, the aldehydes **88a**–**d** were obtained in high yields and good purity. The reaction of aldehydes **88a**–**d** with NH_2_OH**·**HCl gave rise to the corresponding oximes which, upon treatment with NaOCl in the presence of Et_3_N, afforded the substituted isoxazoline derivatives **89a**–**d**, respectively, as a mixture of diastereomers (Scheme 23). Similarly, the reaction of aldehydes **84a**–**c** with propargyl bromide in dry DMF using Cs_2_CO_3_ and the deprotection of the acetal moiety in the presence of AcOH/H_2_O resulted in the formation of aldehydes **90a**–**c** (Scheme 23). The transformation of **90a**–**c** was achieved by their reaction with NH_2_OH**·**HCl and with NaOCl to produce the required isoxazole derivatives **91a**–**c**.

In order to further diversify the range of the products which could be generated by applying intramolecular 1,3-dipolar cycloaddition reaction, the aldehydes **90a**–**c** were treated with sarcosine in dry toluene under refluxing conditions. This reaction yielded the β-carboline-fused pyrroles **92a**–**c** (Scheme 23).

**Scheme 24:**
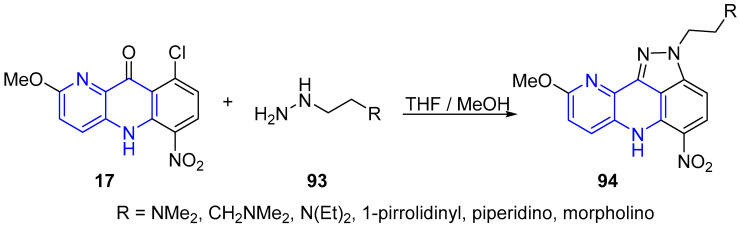
Condensation reaction of chloro benzo[*b*][1,5]naphthyridinone with hydrazines.

A series of dihydroindazolo[4,3-*bc*][1,5]naphthyridines **94** was prepared by condensation of 9-chloro-2-methoxy-6-nitro-5,10-dihydrobenzo[*b*][1,5]naphthyridin-10-one **17** (previously prepared Scheme 5, vide supra) [15]. Thus, condensation of **17** with appropriate (ω-aminoalkyl) hydrazines **93** in tetrahydrofuran/methanol (1:1) at room temperature afforded the desired 2,6-dihydroindazolo[4,3-*bc*][1,5]naphthyridine **94** (Scheme 24).

**Scheme 25:**
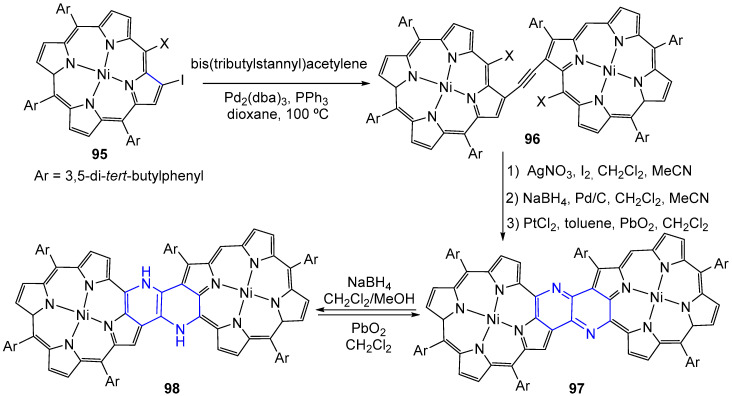
Synthesis of fused 1,5-naphthyridine porphyrin dimers.

Recently [39], starting from 2-iodo-5,10,15-tris(3,5-di-*tert*-butylphenyl)porphyrin niquel(II) complex **95** (Scheme 25), β-to-bethynylene-bridged Ni^II^ porphyrin dimer **96** was obtained by a two-fold Stille coupling reaction with a half equivalent of bis(tributylstannyl)acetylene.

Nitration of **96** with AgNO_3_ and I_2_, hydrogenation by using Pd/C, NaBH_4_, and then Pt^II^-catalyzed intramolecular cyclization with a catalytic amount of PtCl_2_ and oxidation with PbO_2_ afforded the 1,5-naphthyridine-fused porphyrin dimer **97**. In the next step, the dimer **97** was reduced with an excess of NaBH_4_ to give dimer **98** (Scheme 25). This product is quite electron-rich owing to the presence of a 1,2-diaminoethene bridge and oxidizes back to **97** within several hours in solution under ambient conditions. Furthermore, treatment of **98** with PbO_2_ cleanly afforded **97**.

**Figure 5:**
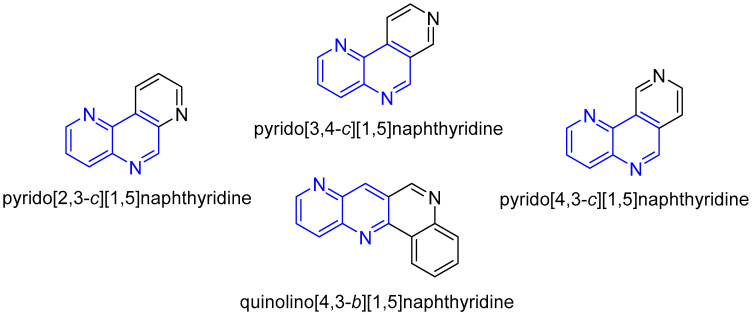
Examples of fused 1,5-naphthyridines with six-membered nitrogen heterocycles.

Pyridine and quinoline ring systems are heterocycles with a wide range of synthetic and medicinal applications [40,41,42,43,44]. For this reason, fused pyrido- and/or quinolino-naphthyridines (Figure 5) may be considered as very interesting and useful nitrogenated heterocycles.

**Scheme 26:**
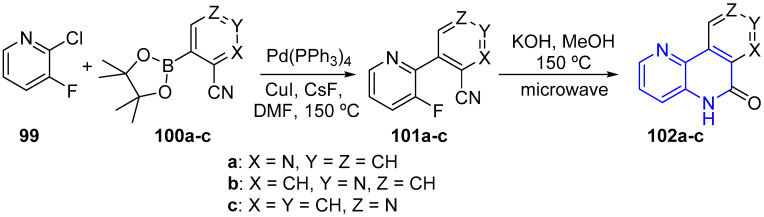
Preparation of pyrido[4,3 or 3,4 or 2,3-*c*][1,5]naphthyridinones.

The pyridonaphthyridines, are difficult to chemical access, but pyrido[4,3 or 3,4 or 2,3-*c*][1,5]naphthyridines were obtained with good yields after chlorodehydroxylation and dehalogenation reactions starting from the parent pyridonaphthyridinones [45]. These pyridonaphthyridinones **102a**–**c** were synthesized (Scheme 26) in a two-step procedure using a Suzuki cross-coupling reaction between 2-chloro-3-fluoropyridine **99** and *ortho-*cyanopyridylboronic esters **100a**–**c** followed by a KOH-mediated anionic ring closure which was performed under microwave heating conditions. The three expected pyrido[*c*][1,5]naphthyridin-6-ones **102a**–**c** were isolated in good yield (Scheme 26).

**Scheme 27:**
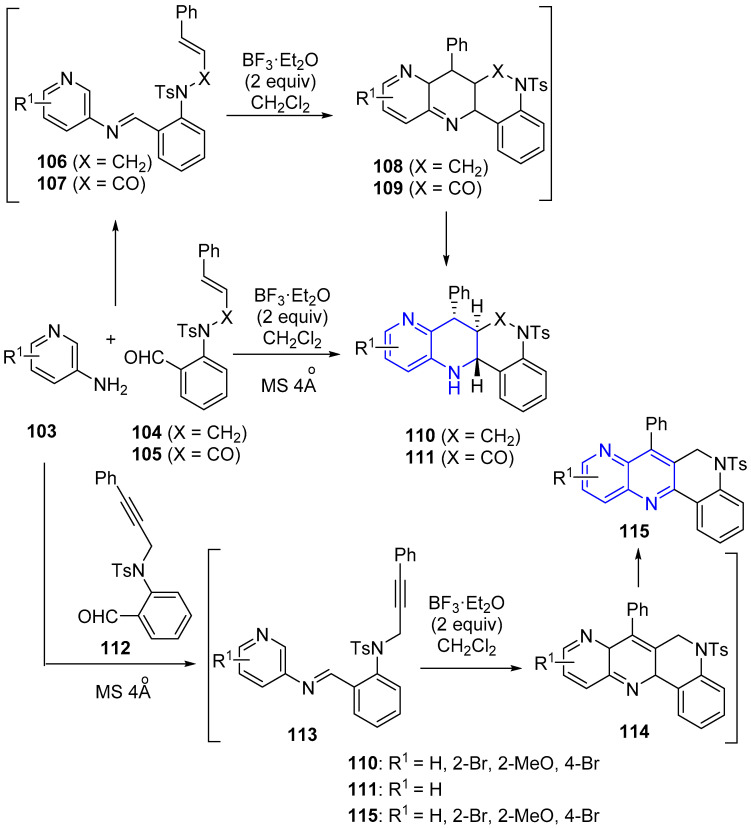
Intramolecular Povarov synthesis of quinolino[4,3-*b*][1,5]naphthyridine derivatives.

The synthesis of quinolino[4,3-*b*][1,5]naphthyridines and quinolino[4,3-*b*][1,5]naphthyridin-6(5*H*)-ones was described recently [46]. The hybrid substituted quinolino[4,3-*b*][1,5]naphthyridines were prepared by an intramolecular Povarov [4+2]-cycloaddition reaction of functionalized aldimines **106** (X = CH_2_) or **107** (X = CO), obtained by the condensation of 3-aminopyridines **103** and unsaturated aldehydes **104** (X = CH_2_) or **105** (X = CO), respectively, in refluxing chloroform in the presence of Lewis acid as BF_3_·Et_2_O (Scheme 27). In this sense, the corresponding tetracyclic *endo*-5-tosyl-5,6,6a,7,12,12a-hexahydroquinolino[4,3-*b*] [1,5]naphthyridines **110** (X = CH_2_) or *endo-*5-tosyl-6a,7,12,12a-tetrahydroquinolino[4,3-*b*] [1,5]naphthyridin-6(5*H*)-ones **111** (X = CO) were selectively obtained with good yields (Scheme 27). Formation of derivatives **110** and **111** may be explained by a regio- and stereospecific intramolecular [4+2]-cycloaddition reaction of aldimines **106/107** to give intermediates **108**/**109** followed by prototropic tautomerization. The methodology tolerates a wide range of electron-releasing and electron-withdrawing substituents in the starting 3-aminopyridines **103**.

The preparation of 5-tosyldihydroquinolino[4,3-*b*][1,5]naphthyridines **115** (Scheme 27) in a single step may be also performed. When aldehyde **112** with a triple bond in their structure reacts with 3-aminopyridines **103** would give the corresponding aldimines **113**, which after a subsequent intramolecular cycloaddition in the presence of BF_3_·Et_2_O yielded the 5-tosyldihydroquinolino[4,3-*b*] [1,5]naphthyridines **115** with good yields. Formation of 5-tosylquinolino[4,3-*b*][1,5]naphthyridine derivatives **115** may be explained in a similar way that previously, by a regiospecific intramolecular [4+2]-cycloaddition reaction of aldimines **113** to give intermediates **114** followed by protodehydrogenation under the reaction conditions (Scheme 27).

### 2.3. Synthesis of 1,5-Naphthyridines Fused with Oxygen-Containing Heterocycles

**Figure 6:**
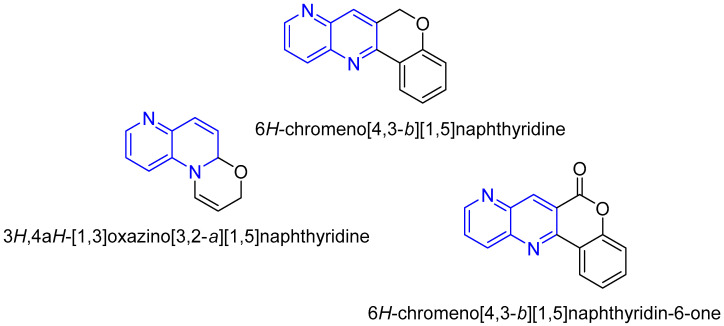
Examples of fused 1,5-naphthyridines with oxygen-containing heterocycles.

The low toxicity of the natural products containing chromene and their broad pharmacological properties are attractive feature for medicinal chemists and a source of inspiration for the design of novel therapeutic agents [47,48]. Therefore, naphthyridines fused with 1,3-oxazines, chromenes or chromenones are very interesting substrates in the search of new drug candidates (Figure 6).

**Scheme 28:**
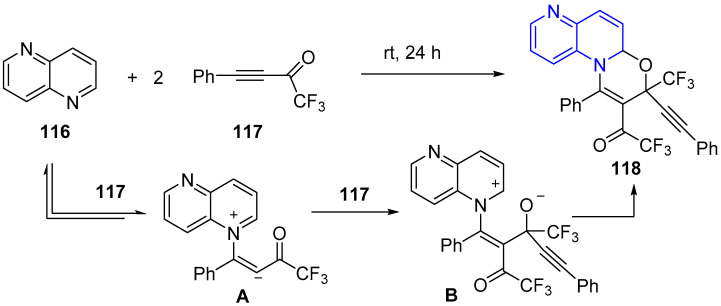
Preparation of [1,3]oxazino[3,2-*a*][1,5]naphthyridine.

Metal- and solvent-free reaction of 1,5-naphthyridine **116** with two molecules of phenyltrifluoroacetylacetylene **117** afforded 3,4a-dihydro-[1,3]oxazino[3,2-*a*][1,5]naphthyridine **118** (Scheme 28). So, when reactants **116** and **117** were allowed to contact at room temperature in the absence of water and solvent, compound **116** appeared to be capable of assembling with two molecules of trifluoroacetylacetylene **117** to form 3,4a-dihydro-[1,3]oxazino[3,2-*a*][1,5]naphthyridine **118** [49]. The authors propose a mechanism in which the first step is the reversible formation of the intermediate 1,3-dipole **A**. Subsequent addition of the second molecule of acetylene **117** proceeds selectively to its carbonyl group. Afterwards, the oxygen center of formed anion **B** attacks position 2 of the naphthyridine ring to cause the product **118** (Scheme 28). The suggested mechanism is in agreement with the experimental results. Indeed, the reversible formation of 1,3-dipole complex **A** is supported by the fact that at a higher temperature, yields of the target products are not improved.

**Scheme 29:**
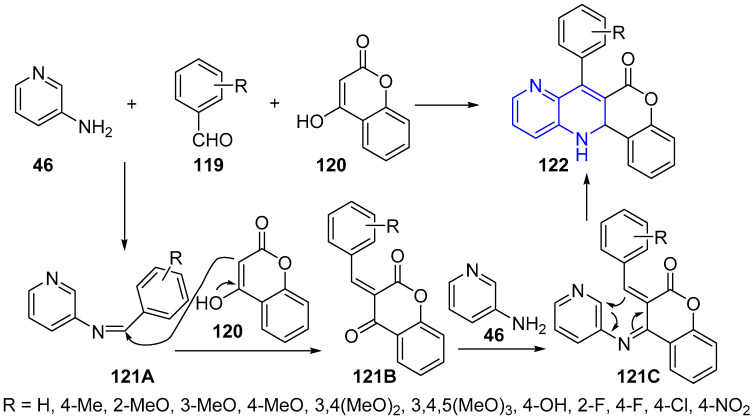
A three-component synthesis of chromeno[4,3-*b*][1,5]naphthyridine derivatives.

A simple and efficient synthesis of chromeno[4,3-*b*][1,5]naphthyridine derivatives **122** (Scheme 29) was accomplished in high yields via the one-pot three-component reaction of 3-aminopyridine **46**, arylaldehydes **119** and 4-hydroxycoumarine **120** in aqueous media catalyzed by sulfamic acid and at 100 °C [50]. The process gave the derivatives **122** in high yields. The nature of substituents on aromatic ring did not show obvious effects in terms of yields. In the mechanism proposed by the authors, first, an imine **121A** is formed via condensation of aldehyde **119** with 3-aminopyridine **46** (Scheme 29). Then, Povarov reaction of 4-hydroxycoumarin moiety with the imine **121A** gave a cyclized intermediate **121C**. Subsequently, the double bond isomerization furnishes the final product **122**.

**Scheme 30:**
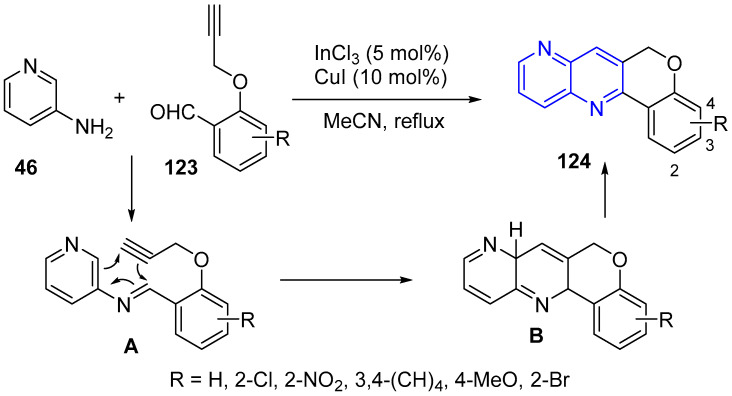
A domino reaction for the synthesis of chromeno[4,3-*b*][1,5]naphthyridine derivatives.

A mild and efficient method for the synthesis of chromenonaphthyridine derivatives via domino reaction of 3-aminopyridine **46** and different *O*-propargylated salicylaldehydes **123** (Scheme 30) using CuI/InCl_3_ as an efficient catalyst, refluxed in acetonitrile have been reported [51]. Mild reaction conditions, operational simplicity, good-to-excellent yield, and easy isolation of product is the silent feature of this reaction to afford the corresponding 6*H*-chromeno[4,3-*b*][1,5]naphthyridine derivatives **124** in high yields (Scheme 30). Different *O*-propargylated salicylaldehydes **123**, containing electron-withdrawing and electron-donating substituents, exhibited equal activity towards the formation of product in good to excellent yields. Plausible mechanistic rationalization for the formation of chromenonaphthyridine derivatives **124** is depicted in Scheme 30. Initially, imine **A** is formed which contained the aza-heterodiene moiety. This aza-heterodiene undergoes intramolecular aza Diels-Alder reaction with the propargyl triple bond, which is activated by indium chloride followed by aromatization to give the desired products **124**.

**Scheme 31:**
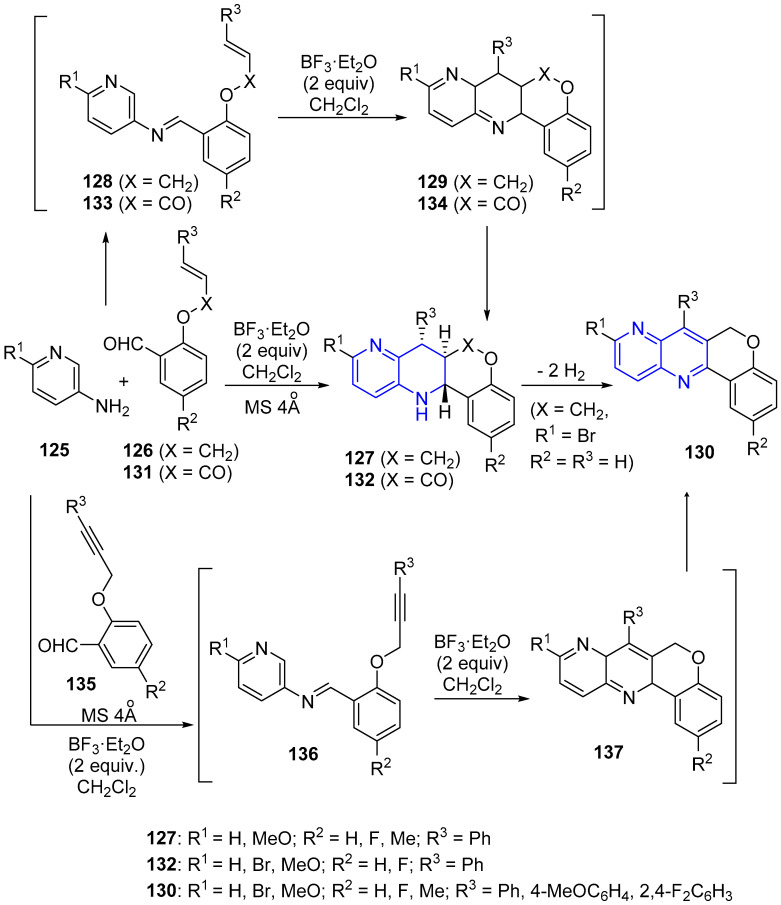
Intramolecular aza-Diels-Alder reaction to obtain hybrid, fused naphthyridines.

Hybrid tetrahydro[1,5]naphthyridine and [1,5]naphthyridine derivatives fused with heterocycles such as chromenes and chromen-2-ones or coumarins were synthesized in good to high general yields [52]. The synthetic route involves an intramolecular [4+2] cycloaddition reaction of functionalized aldimines and aldehydes containing a double or triple carbon-carbon bond in *ortho* position and allows the selective generation of three stereogenic centers in a short, efficient and reliable synthesis (Scheme 31). Aldimines **128** (X = CH_2_), prepared in situ by condensation reaction of 3-aminopyridines **125** and previously prepared functionalized aldehydes **126** (X = CH_2_), cyclized intramolecularly in refluxing chloroform and in the presence of BF_3_·Et_2_O (Scheme 31) to give **129** by a regio- and stereospecific intramolecular [4+2]-cycloaddition reaction which after prototropic tautomerization afforded compounds **127**. When 2-(allyloxy)benzaldehyde **126** (R^2^ = R^3^ = H) and 6-bromo-3-aminopyridine **125** (R^1^ = Br) were used, after formation of the corresponding tetrahydro[1,5]naphthyridine **127**, subsequent dehydrogenation under the reaction conditions gave dehydrogenated derivative **130** (Scheme 31).

In order to increase the molecular diversity, the methodology was extended to the preparation of angularly fused tetracyclic derivatives **132** (X = CO), in which the chromene scaffold was substituted by a coumarin (chromen-2-one). As before, functionalized aldehydes **131** (Scheme 31, X = CO) were condensed with 3-aminopyridines **125** to give aldimines **133** (X = CO). These imines **133** cyclized intramolecularly in refluxing chloroform in the presence of two equivalents of Lewis acid (BF_3_·Et_2_O) to afford *endo*-6a,7,12,12a-tetrahydro-6*H*chromeno[4,3-*b*][1,5]naphthyridin-6-ones **132** (X= CO) with good yields in a regio- and stereospecific way (Scheme 31). The formation of these polycyclic compounds **132** may be explained, as before, by a regio- and stereospecific intramolecular [4+2]-cycloaddition reaction of aldimines **133** (X = CO) to give intermediates **134** (X =CO) followed by prototropic tautomerization. The methodology tolerates electron-releasing and electron-withdrawing substituents in the aromatic aldehydes, even fluorinated ones that allow the preparation of fluoro containing compounds, interesting substrates from a biological point of view.

If acetylenes are used as dienophiles instead of olefins, the corresponding 1,5-naphthyridine compounds **130** may be directly obtained. The formation of these chromeno[4,3-*b*][1,5]naphthyridines **130** can be performed by initial condensation reaction between 3-aminopyridine **125** and functionalized aldehydes **135** containing an alkyne group in the *ortho* position. Subsequent intramolecular [4+2]-cycloaddition reaction in refluxing chloroform, in the presence of two equivalents of BF_3_·Et_2_O, would give intermediates **137** followed by dehydrogenation under the reaction conditions to afford chromeno[4,3-b][1,5]naphthyridines **130** with good yields (Scheme 31).

### 2.4. Synthesis of 1,5-Naphthyridines Fused with Sulphur-Containing Heterocycles

**Scheme 32:**
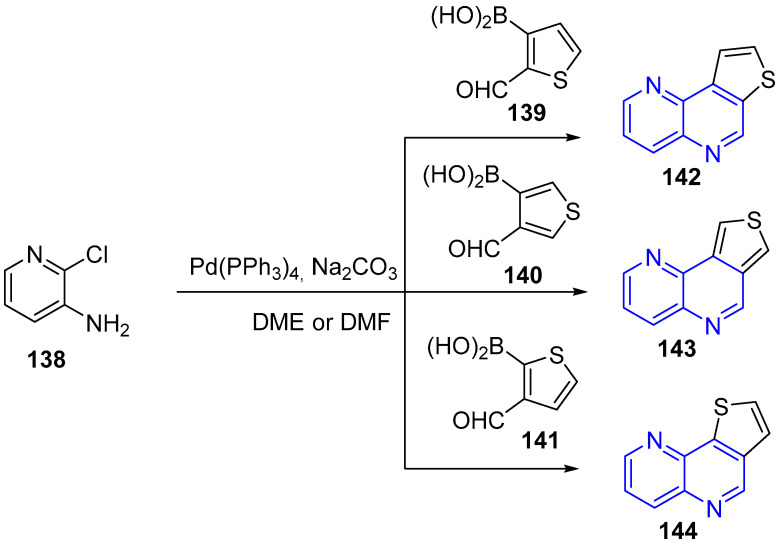
Palladium-catalyzed synthesis of thieno[1,5]naphthyridines.

A one-stage procedure has been collected by Litvinov et al. for the preparation of isomeric 1,5-naphthyridines fused with a thiophene ring [4]. The procedure used Pd(PPh_3_)_4_-catalyzed cross-coupling of 2-chloropyridin-3-amine **138** with thiopheneboronic acids **139**–**141** (Scheme 32) containing an *ortho*-formyl group. The cross-coupling products cyclized spontaneously during the reaction to give thieno[1,5]naphthyridines **142**–**144** with all possible types of ring fusion. The effects of the catalyst amount, the nature of the base, and the reaction time on the yield of the naphthyridine **143** have been studied.

**Scheme 33:**
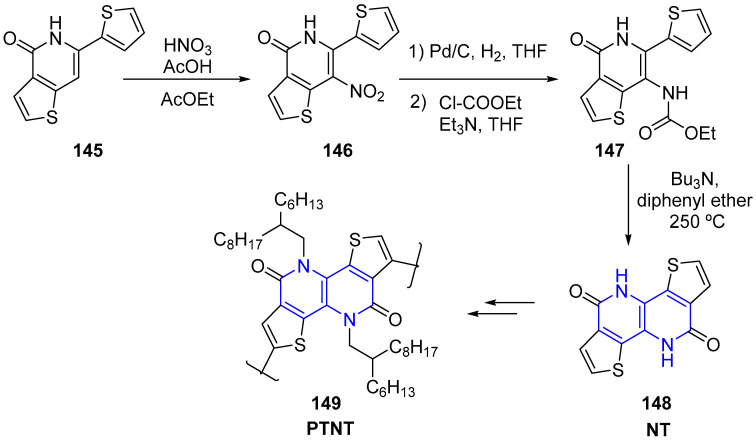
Preparation of dithieno[3,2-*c*:3,2-*h*][1,5]naphthyridine NT, a precursor of PTNT.

A synthetic route previously reported by Bisagni et al. [53] was modified [54] for the synthesis of 4,9-bis(2-hexyldecyl)-4,9-dihydrodithieno[3,2-*c*:3,2-*h*][1,5]naphthyridine-5,10-dione **148** (NT) and the poly(2,5-thiophene-4,9-bis(2-hexyldecyl)-4,9-dihydrodithieno[3,2-*c*:3,2-*h*][1,5]naphthyridine—5,10-dione **149** (PTNT, Scheme 33). Nitration of derivative **145** at low temperature seemed to be selective for the proton on the 7-position and offered **146**. Reduction with hydrogen/Pd/C and subsequent reaction with ethyl chloroformate offered the derivative with a carbamate group **147**. Subjecting **147** to high temperatures caused nucleophilic attack of the thiophene to the carbonyl, after which the NT unit **148** was obtained.

## 3. Reactivity of Fused 1,5-Naphthyridines

### 3.1. N-Alkylation

The *N*-alkylation of the fused dihydro- or tetrahydro[1,5]naphthyridines is one of the reactions frequently carried out in the fused system. They are generally S_N_ reactions on alkyl halides or reductive alkylation reactions.

**Scheme 34:**
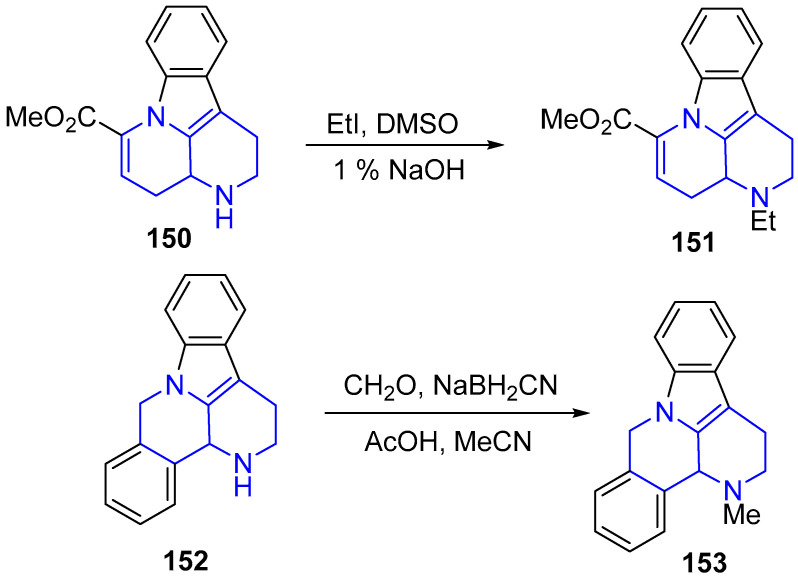
*N*-alkylation of fused 1,5-naphthyridines.

Methyl 3-ethyl-2,3,3a,4-tetrahydro-1*H*-indolo[3,2,1-*de*][1,5]naphthyridine-6-carboxylate **151** (Scheme 34) was prepared by the *N*-alkylation of ester **150** with iodoethane in DMSO [4]. In a similar way, 2,3,9,13b-tetrahydro-1*H*-benzo[*c*]indolo[3,2,1-*ij*][1,5]naphthyridine **152** (Scheme 34) has been used in the synthesis of **153** [4]. Thus, the reductive methylation of **152** with formaldehyde and sodium cyanoborohydride as a source of hydride gives compound **153** (Scheme 34).

**Scheme 35:**
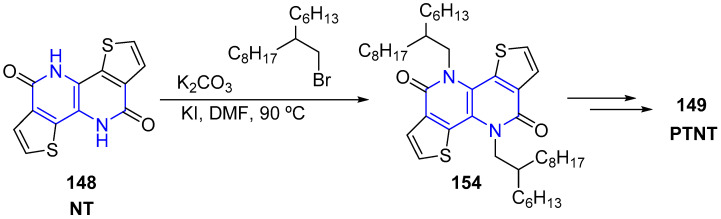
*N*-alkylation of dithieno[3,2-*c*:3,2-*h*][1,5]naphthyridine **NT**.

NT, (4,9-bis(2-hexyldecyl)-4,9-dihydrodithieno[3,2-c:3,2-*h*][1,5]naphthyridine-5,10-dione) **148**, can be *N*-alkylated (Scheme 35) with 2-hexyldecyl bromide to give **154** the necessary compound in the polymerization of NT to obtain PTNT polymer **149** [54].

### 3.2. Electrophilic Substitution Reactions (S_E_Ar)

**Scheme 36:**
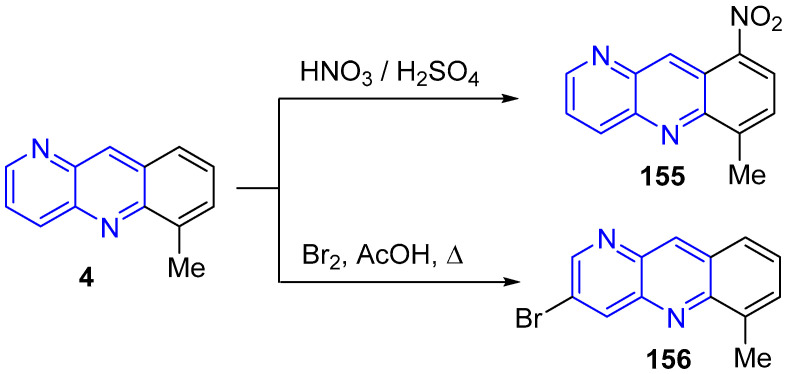
Nitration and bromination of benzo[*b*][1,5]naphthyridines.

It is known that the nitration of benzonaphthyridines with a HNO_3_/H_2_SO_4_ mixture occurs exclusively in the benzene ring. Thus, nitration of 6-methylbenzo[*b*][1,5]naphthyridine **4** gives the corresponding nitro derivative **155** (Scheme 36). In these reactions, the nitro group is also attached to the benzene ring in the *para* position with respect to the methyl substituent [5].

At the same time, 6-methylbenzo[*b*]naphthyridines are brominated at the peripheral pyridine ring in the β-position with respect to the nitrogen atom (rather than at the benzene ring). This reaction pathway cannot be explained in terms of the electrophilic substitution mechanism (aromatic SE (AE)). In this case, the behavior of benzonaphthyridine **4** (Scheme 36) is similar to that of quinoline and isoquinoline, when its bromination in weakly acidic media also occurs at the pyridine ring in the β-position with respect to the nitrogen atom giving **156**, rather than at the benzene ring [5]. The mechanism for the bromination of these compounds entails nucleophilic addition-elimination [S_N_(AE)].

**Scheme 37:**
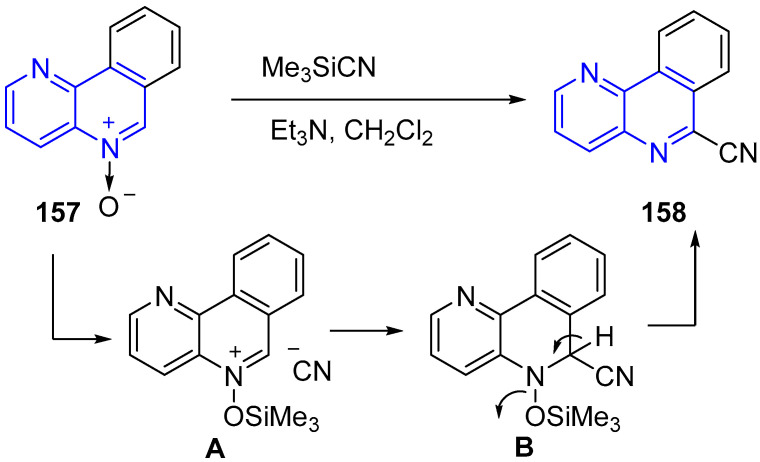
Cyanation of benzo[*c*][1,5]naphthyridine by means of *N*-oxide derivative.

More recently, the synthesis of benzo[*c*][1,5]naphthyridine-6-carbonitrile **158**, starting from benzonaphthyridine *N*-oxide **157** [55], has been achieved following the methodology of cyanation of two unsubstituted 1*H*-imidazole 3-oxides [56]. Benzo[*c*][1,5]naphthyridine-5-oxide **157** in dry CH_2_Cl_2_ in the presence of Et_3_N was treated with Me_3_SiCN at 0–5 °C and benzo[c][1,5]naphthyridine-6-carbonitrile **158** was obtained in good yield. The reaction mechanism for the formation of compounds **158** is analogous to that proposed for the cyanation of azine *N*-oxides, silylation of the *N*-oxide leads to the benzonaphthyridinium ion **A**, which adds a cyanide ion to give the intermediate **B** and which, in turn, eliminates Me_3_SiOH (Scheme 37).

### 3.3. Nucleophilic Substitution Reactions (S_N_Ar)

**Scheme 38:**
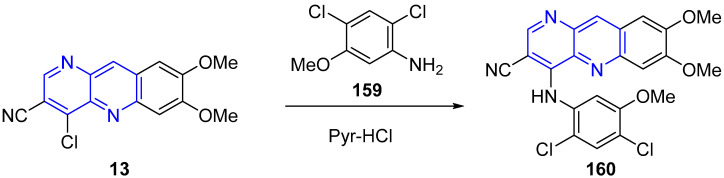
S_N_Ar at position 4 of benzo[*b*][1,5]naphthyridine ring.

One of the major procedures used in the synthesis of biologically active fused 1,5-naphthyridines is based on the replacement (S_N_Ar) of the halogen atom at position 4 of the benzo[*b*][1,5]naphthyridine ring. Thus, coupling of **13** (Scheme 38) with 2,4-dichloro-5-methoxyaniline **159** gave target compound **160** [4].

**Scheme 39:**
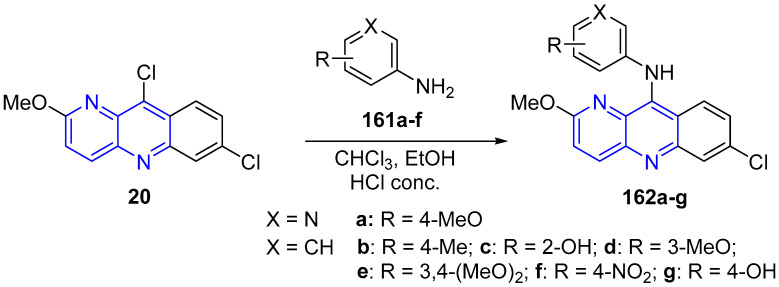
S_N_Ar at position 10 of benzo[*b*][1,5]naphthyridine ring.

Similarly, the replacement (S_N_Ar) of the halogen atom at position 10 of the benzo[*b*][1,5]naphthyridine system occurred. The reaction of **20** with aniline derivatives **161** in the presence of a few drops of concentrated hydrogen chloride (Scheme 39) afforded the amino derivatives benzo[*b*][1,5]naphthyridines **162a**–**e** [15] or **162f** [57]. The same reaction was used in the industrial preparation of pyronaridine tetraphosphate **21** (Scheme 6, vide supra) a well-known antimalarial drug. Thus, the halogen atom at position 10 of the benzo[*b*][1,5]naphthyridine ring in **20** was replaced (S_N_Ar) by an aminophenol group to give compound **162g** (Scheme 39) used later in the preparation of pyronaridine tetraphosphate [17].

**Scheme 40:**
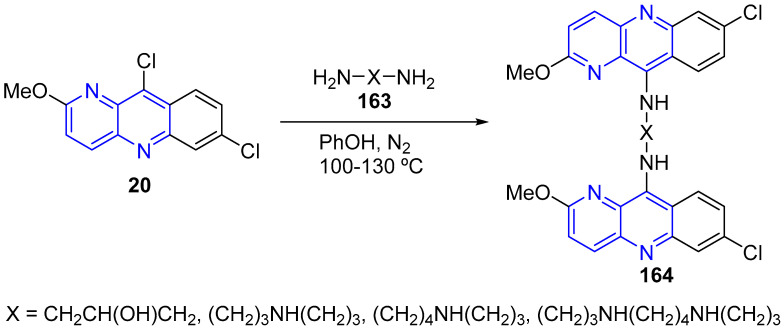
Double S_N_Ar using diamines for the synthesis of dimeric benzo[*b*][1,5]naphthyridines.

The same procedure, a double S_N_Ar, if diamines are used can lead to dimers. Thus, dimeric benzo[*b*][1,5]naphthyridines **164** (Scheme 40) were synthesized by the S_N_Ar reaction of 7,10-dichlorobenzonaphthyridine **20** with di- and polyamines **163** [5].

**Scheme 41:**
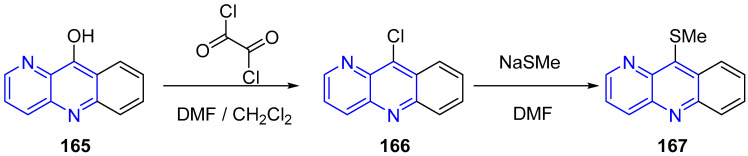
Methanethiolation of benzo[*b*][1,5]naphthyridine.

The reactivity of position 10 of the benzo[*b*][1,5]naphthyridine system has been used for the synthesis of other functionalized derivatives with a benzo[*b*][1,5]naphthyridine unit [58]. Benzo[*b*][1,5]naphthyridin-10(5*H*)-one **165** (Scheme 41) was converted into the corresponding chloro derivative **166**, using the Vilsmeier reagent generated from oxalyl chloride and DMF (Scheme 41). Subsequent reaction with methanethiolate provided **167**.

In the preparation of 2,6-dihydroindazolo[4,3-*bc*][1,5]naphthyridine **94** (Scheme 24, vide supra), the condensation of 9-chloro-2-methoxy-6-nitro-5,10-dihydrobenzo[*b*][1,5]naphthyridine-10-one **17** (previously prepared) with the appropriate (ω-aminoalkyl)hydrazine **93** in tetrahydrofuran/methanol (1:1) at room temperature proceeds through an intramolecular S_N_Ar of the halogen atom at position 9 giving to desired compounds **94** [15].

**Scheme 42:**
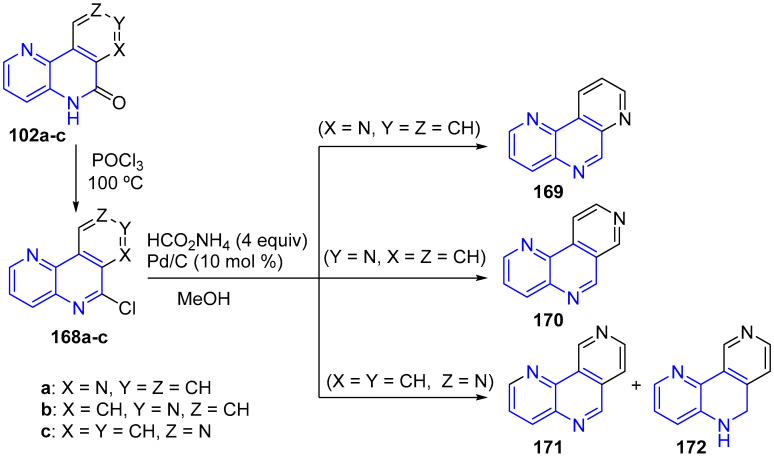
Preparation of pyrido[*c*][1,5]naphthyridines from pyrido[*c*][1,5]naphthyridin-6-ones.

Otherwise, chlorodehydroxylation reaction of three pyrido[*c*][1,5]naphthyridin-6-ones **102a**–**c** (Scheme 42) was carried out by using phosphorus oxychloride at 100 °C for 12h and the 6-chloropyrido[*c*][1,5]naphthyridines **168a**–**c** were easily obtained in good yields [45]. Subsequently, derivatives **168a**–**c** (Scheme 42) were submitted to a dehalogenation with four equivalents of ammonium formate and palladium on charcoal in methanol. Reacting 6-chloropyrido[2,3-*c*][1,5]naphthyridine **168a** (X = N, Y = Z = CH) to reflux, the total conversion into 1,5-naphthyridine **169** was observed. Similarly, compound **168b** was submitted to the same conditions and at this time the reaction afforded the pyrido[3,4-*c*][1,5]naphthyridine **170** in a high yield. Finally, the 1,5-naphthyridine **168c** after 12 h of stirring in MeOH, showed a total conversion and the formation of two inseparable compounds: the expected compound **171** and a hydrogenated 5,6-dihydropyrido[4,3-*c*]-1,5-naphthyridine **172** (Scheme 42).

### 3.4. Oxidation and Reduction

**Scheme 43:**
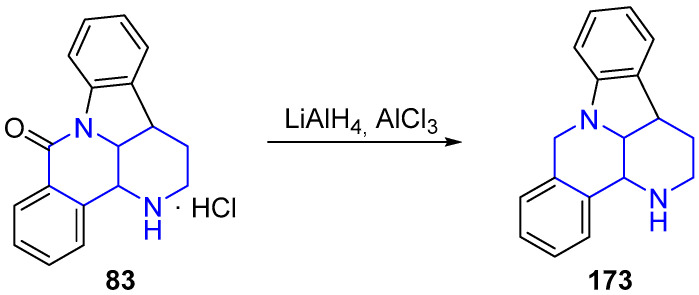
Hydride reduction of benzo[*c*]indolo[3,2,1-*ij*][1,5]naphthyridin-9(2*H*)-one.

The most common reduction reaction of 1,5-naphthyridine system fused with carbocycles or heterocycles is the transformation of naphthyridinones into naphthyridines or what is the same, the transformation of the carbonyl group into methylene group in different conditions. For instance in Reference [4], tetrahydro-1*H*-benzo[*c*]indolo[3,2,1-*ij*][1,5]naphthyridin-9(2*H*)-one hydrochloride **83** previously obtained (Scheme 22, vide supra) gave the reduced compound **173** by treatment with LiAlH_4_ and AlCl_3_ (Scheme 43).

**Scheme 44:**
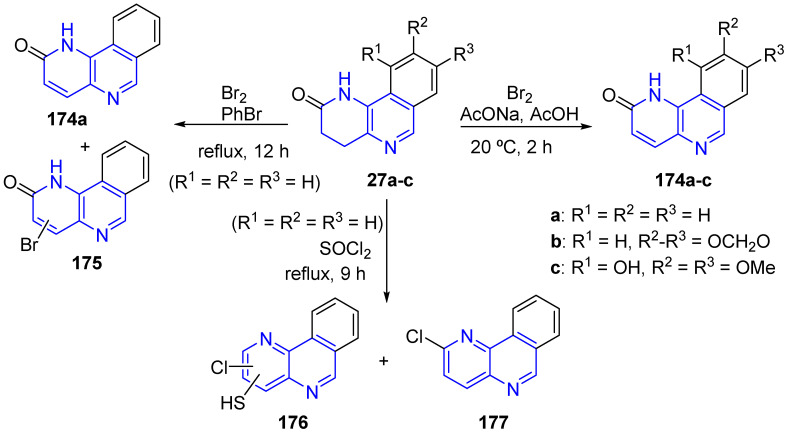
Oxidation reactions of 1,2,3,4-tetrahydrobenzo[*c*][1,5]naphthyridin-2(1*H*)-ones.

However, the most usual is the oxidation (dehydrogenation) of tetrahydro- or dihydro[1,5]naphthyridines fused with carbocycles to give the corresponding dehydrogenated heterocycles and these transformations can be carried out under different oxidation conditions. The aromatization of compounds **27a**–**c** (Scheme 8, vide supra) under different conditions allowed to obtain 3,4-dihydrobenzo[*c*][1,5]naphthyridin-2(1*H*)-ones **174** and **175** (Scheme 44) or benzo[*c*][1,5]naphthyridines **176** and **177** [18]. So, bromine in AcONa/AcOH led only, in an irreproducible manner, to very few amounts of pyridones **174a**–**c** from lactams **27a**–**c**. However, when lactam **27a** (R^1^ = R^2^ = R^3^ = H) was refluxed with 1.2 equivalents of bromine in bromobenzene for 12 h, pyridine **174a**, accompanied by monobrominated compound **175** (position of the bromine atom was not determined), was obtained. Alternatively, by heating **27a** with thionyl chloride for 9 h, it furnished a mixture of **176** containing a chlorine and a sulphur atom (position of substituents was not determined) and chloronaphthyridine **177** (Scheme 44).

DDQ is generally used for the transformation of hydrogenated compounds into the corresponding aromatic compounds. Thus, the addition of DDQ to the crude reaction mixture of pyrido[4,3-*c*][1,5]naphthyridine **171** and a hydrogenated product **172** (Scheme 42, vide supra), showed a total conversion into pyrido[4,3-*c*][1,5]naphthyridine **171** [45].

**Scheme 45:**
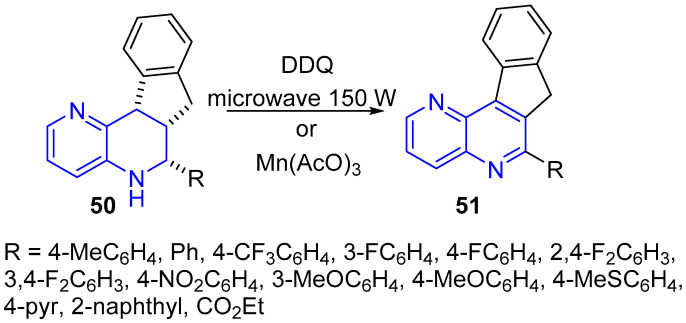
Dehydrogenation of 1,2,3,4-tetrahydroindeno[*c*][1,5]naphthyridines.

The transformation of 1,2,3,4-tetrahydroindeno[1,5]naphthyridine **50** into dehydrogenated derivatives **51** was performed by our group [24,25] by dehydrogenation under microwave irradiation (150 W), with one equivalent of DDQ (Scheme 45) with total conversion of starting material. Alternatively, dehydrogenation of tetrahydroindeno[1,5]naphthyridine **50** with a ethyl carboxylate group (R = CO_2_Et) was performed by using Mn(AcO)_3_ as oxidant in acetic acid at room temperature and the corresponding 7*H*-indeno[2,1-*c*][1,5]naphthyridine **51** (R = CO_2_Et) was obtained.

**Scheme 46:**
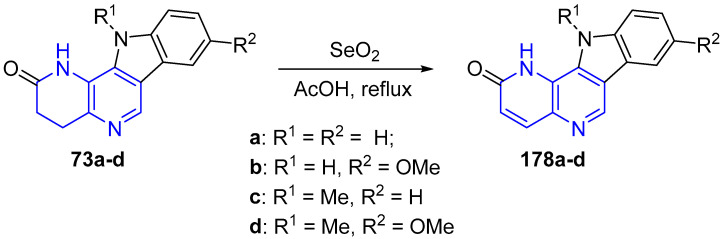
Oxidation of tetrahydro-2*H*-indolo[3,2-c][1,5]naphthyridinones.

On the other hand, dehydrogenation of tetrahydro-2*H*-indolo[3,2-c][1,5]naphthyridin-2-ones **73a**–**d** previously described (Scheme 19, vide supra) to fused naphthyridinones **178a**–**d** (Scheme 46) proceeded with good yields by refluxing them with selenium oxide in acetic acid for 15–36 h [18]..

**Scheme 47:**
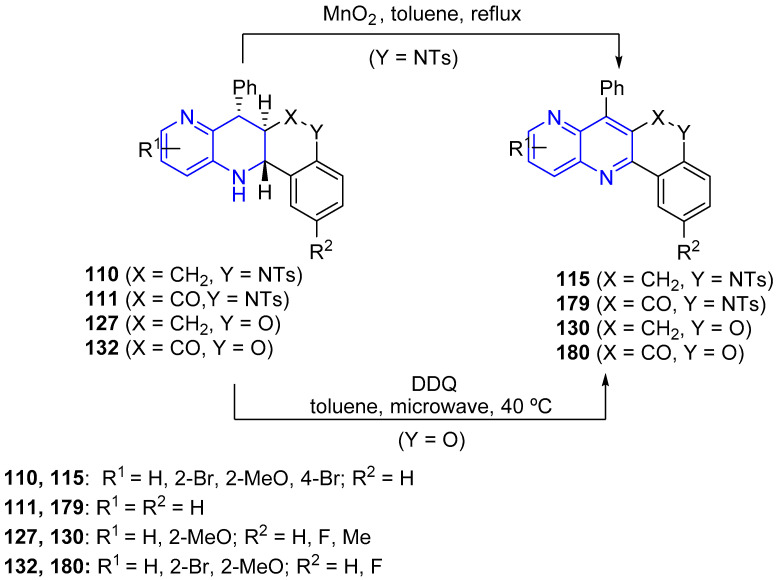
Dehydrogenation of tetrahydro-6*H*-chromeno[4,3-*b*][1,5]naphthyridines.

As recently described [46], the dehydrogenation of **110** (X = CH_2_, Y = NTs) and **111** (X = CO, Y = NTs) with four equivalents of MnO_2_ in toluene at 111 °C for 48 h (Scheme 47) led to the corresponding more unsaturated dihydro[1,5]naphthyridines **115** and [1,5]naphthyridin-6(5*H*)-one derivatives **179** in quantitative yields.

Similarly, the dehydrogenation of tetrahydro-6*H*-chromeno[4,3-*b*][1,5]naphthyridines **127** (X = CH_2_, Y = O, Scheme 47) and naphthyridin-6-ones **132** (X = CO, Y = O, Scheme 47) with 1 equivalent of DDQ in toluene at 40 °C under microwave irradiation for 2 h would afford chromeno[4,3-*b*][1,5]naphthyridines **130** and chromeno[4,3-*b*][1,5]naphthyridin-6-ones **180** (X = CO, Scheme 47) respectively, in quantitative yields [52].

### 3.5. Side Chain Modifications

**Scheme 48:**
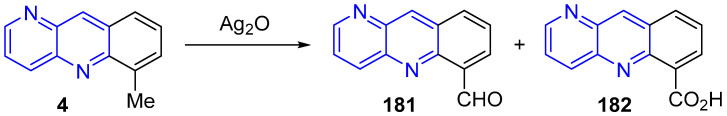
Oxidation of 6-methylbenzo[*b*][1,5]naphthyridine.

There are many modifications of functional groups and side chains that can be made in fused 1,5-naphthyridine derivatives, without altering the 1,5-naphthyridine system. For example, with a basic solution of silver oxide at 20 °C for 30 min, a methyl group in 6-methylbenzo[*b*][1,5]naphthyridine **4** can be oxidized to the formyl group or the carboxyl group (Scheme 48) to get compounds **181** and **182** [4].

**Scheme 49:**
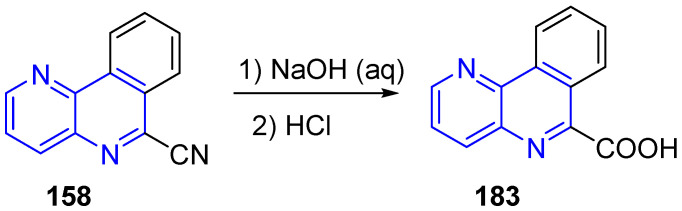
Transformation of nitrile group of benzo[*c*][1,5]naphthyridine-6-carbonitrile.

Similarly, the nitrile group of benzo[*c*][1,5]naphthyridine-6-carbonitrile **158** can also undergo transformations without any modification occurring in the polycyclic system [56]. Thus, compound **158** is hydrolyzed to the corresponding acid **183** by boiling in aqueous alkali (Scheme 49).

**Scheme 50:**
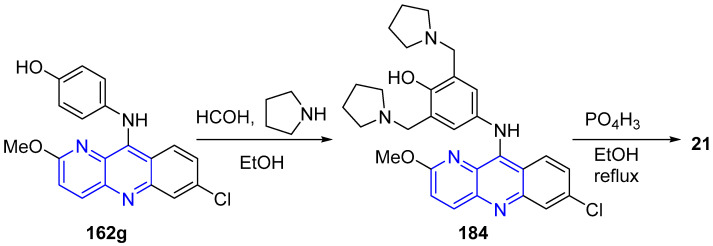
Preparation of pyronaridine tetraphosphate **21**.

In the industrial preparation of antimalarial drug pyronaridine tetraphosphate **21**, the compound **162g** previously described (Scheme 39, vide supra) underwent a Mannich reaction with pyrrolidine in the presence of formaldehyde to give pyronaridine **184** (Scheme 50) and the yield increased about 20% [17]. Finally, **184** was treated with phosphoric acid to yield pyronaridine tetraphosphate **21**.

**Scheme 51:**
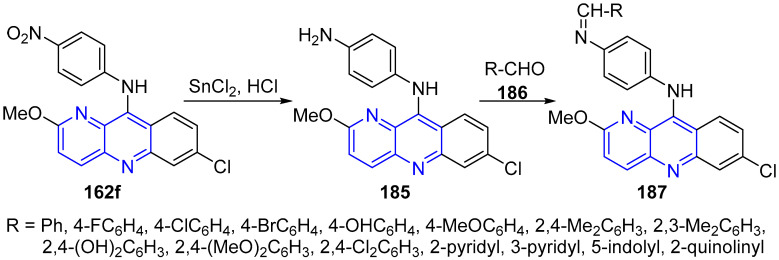
Transformation of the nitro group into imine in a benzo[*b*][1,5]naphthyridine derivative.

The reduction of NO_2_ group in **162f** (Scheme 39, vide supra) using SnCl_2_ as reducing agent (Scheme 51) led to the key intermediate **185** [57]. Then **185** and different aromatic aldehydes **186** were mixed in boiling ethanol affording the target compounds **187** bearing the C=N linkage moiety.

**Scheme 52:**
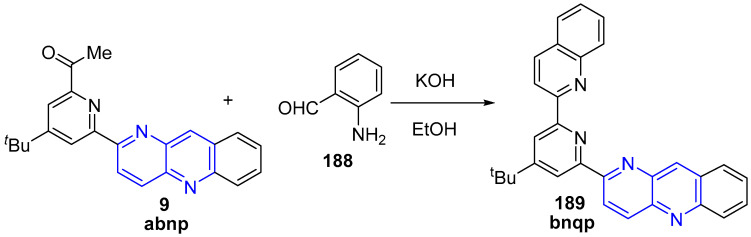
Preparation of tridentate ligand bnqp.

Tridentate ligand 2-(benzo[*b*][1,5]naphthyridin-2-yl)-6-(quinolin-2-yl)-4-tert-butylpyridine **189** (bnqp, Scheme 52) was synthesized by the Friedländer condensation of equimolar amounts of acetyl compound **9** (abnp) and aromatic α-aminoaldehyde **188** in dry ethanol in the presence of KOH [13].

**Scheme 53:**
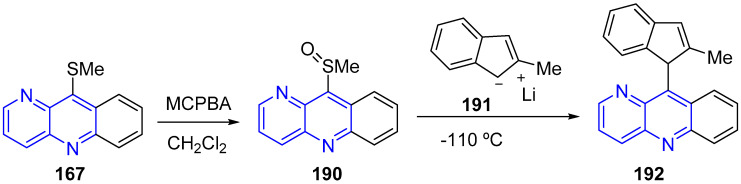
Preparation of the 10-indenylbenzo[*b*][1,5]naphthyridine ligand.

For the synthesis of indenyl ligand functionalized with a benzo[*b*][1,5]naphthyridine unit **192** (Scheme 53) the organosulphide **167** was oxidized to the sulfoxide **190** by using one equivalent of m-chloroperbenzoic acid (MCPBA) [58].The ligand-coupling reaction was carried out using lithium-2-methylindenide (Li-2-MeInd) **191**, the 2-methyl substituent being used to raise the barrier to rotation around the heteroaryl−indene bond. To isolate the 1-heteroaryl-1*H*-indene tautomer, the reaction was carried at −110 °C with 1.3 equivalents of **191** (Li-2-MeInd) for only 30 min. Then, the 1-heteroaryl-1*H*-indene tautomer **192** was isolated (Scheme 53).

**Scheme 54:**
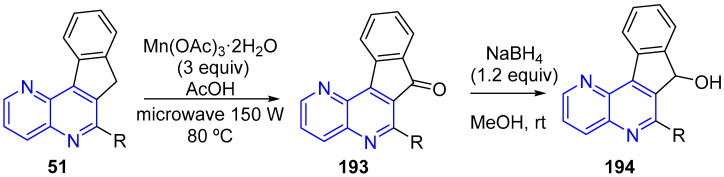
Oxidation and reduction reactions over indeno[1,5]naphthyridines.

The presence of hydroxyl groups in the heterocyclic molecules may offer some advantages such as the enhancement of the solubility of such molecules in the physiological media or the formation of hydrogen bonds with the surrounding amino acids present in the target protein. Reaction between 7*H*-indeno[1,5]naphthyridines **51** with three equivalents of Mn(OAc)_3_ in a microwave reactor in acetic acid (Scheme 54) generated indeno[1,5]naphthyridin-7-ones **193** [24]. The results demonstrated that a wide range of substituents with electron-donating and -withdrawing groups participated in this process. After, by the hydride reduction of carbonyl group present in 7*H*-indeno[1,5]naphthyridine-7-ones **193** with NaBH_4_ in methanol at room temperature the corresponding 7*H*-indeno[2,1-c][1,5]naphthyridin-7-ols **194** were quantitatively obtained (Scheme 54).

**Scheme 55:**
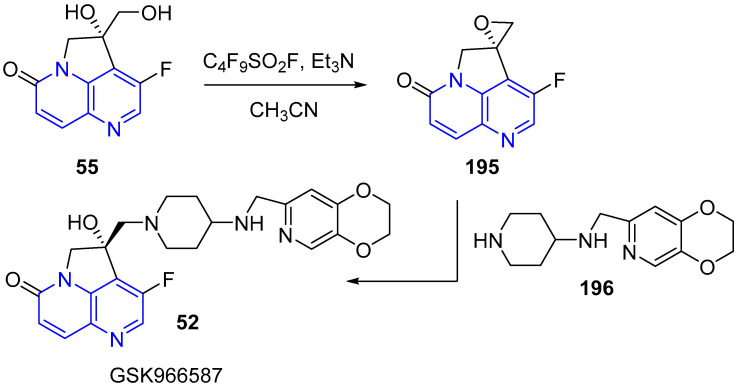
Enantioselective synthesis of the antibiotic GSK966587.

To complete the efficient enantioselective total synthesis of the potent antibiotic GSK966587 **52** (Scheme 16, vide supra), diol **55** was first converted to spiro-epoxide **195** (Scheme 55) using Et_3_N and perfluorobutanesulfonyl fluoride [27]. After 1 h at room temperature and filtration through silica gel, epoxide **195** was treated with functionalized piperidine **196** (1.5 equivalents) at room temperature for 14 h. Afterwards, GSK966587 **52** precipitated directly from the reaction mixture.

**Scheme 56:**
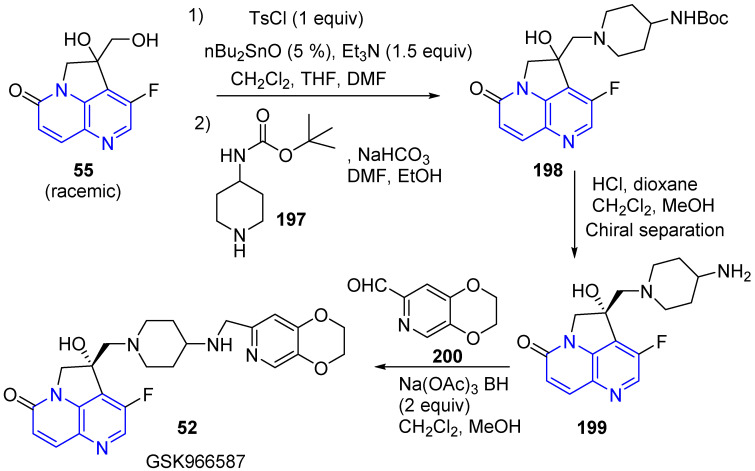
Synthesis of the antibiotic GSK966587 from a racemic diol.

A modification of the above procedure for the total synthesis of **52***,* the antibiotic GSK966587 [28] was the transformation of racemic diol **55** by activation of the primary hydroxyl group as the tosylate using dibutyltin oxide (Scheme 56), reaction with *tert*-butyl piperidin-4-ylcarbamate **197** and displacement with *N*-Boc piperidine to afford the *N*-Boc protected intermediate **198**. The free amine **199** was liberated by Boc deprotection and then separated into enantiomers by prepative chiral HPLC. The desired (4*S*)-enantiomer was placed under reductive alkylation conditions with aldehyde **200** to give **52** (Scheme 56).

**Scheme 57:**
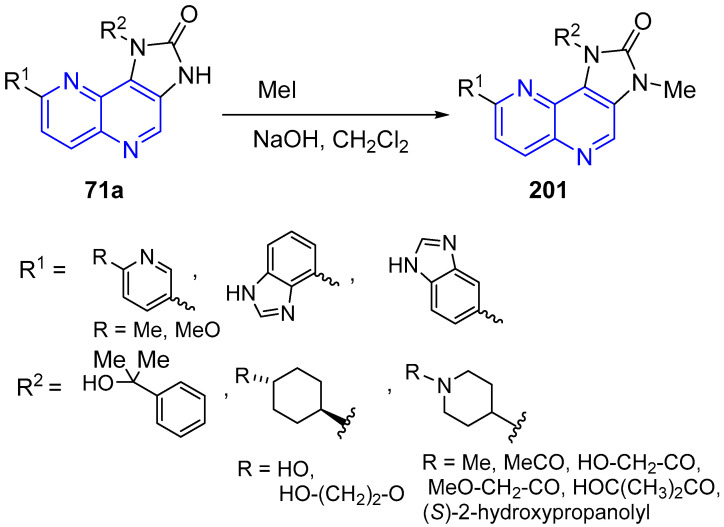
*N*-methylation reaction of 1,3-dihydro-2*H*-imidazo[4,5-*c*][1,5]naphthyridin-2-ones.

In the last stage of the general synthetic route for preparing 3-methyl-1*H*-imidazo[4,5-*c*][1,5]naphthyridin-2(3*H*)-one **201** (Scheme 57), 1,3-dihydro-2*H*-imidazo[4,5-*c*][1,5]naphthyridin-2-ones **71a** (Scheme 18, vide supra) were methylated with MeI in the presence of NaOH to give compound **201** [30].

**Scheme 58:**
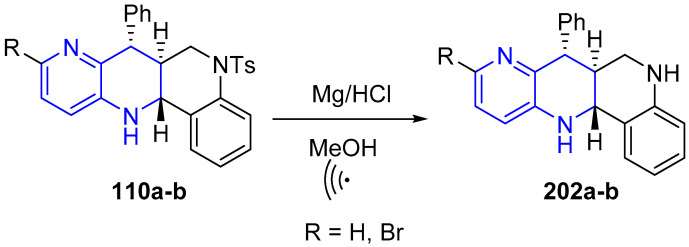
Tosyl deprotection of 5-tosylhexahydroquinolino[4,3-*b*][1,5]naphthyridines.

Recently [46], the deprotection of the tosyl group in compounds **110a**–**b** (R = H, Br) was performed and the corresponding derivatives **202a**–**b** could be isolated when the 5-tosylhexahydroquinolino[4,3-*b*][1,5]naphthyridines **110a**–**b** were treated with Mg under acidic conditions and sonicated for 8 h (Scheme 58).

**Scheme 59:**
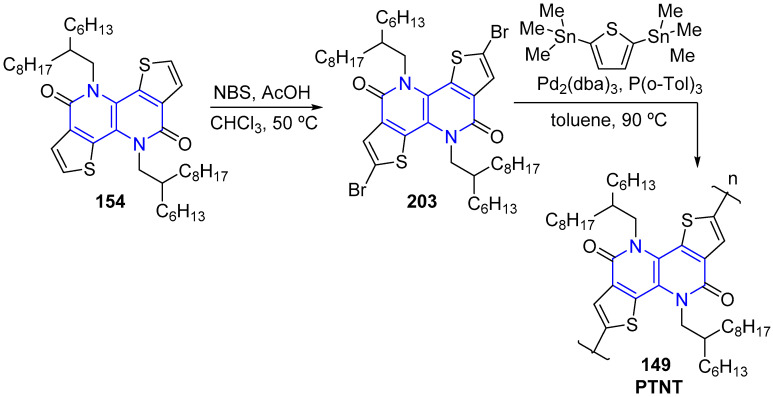
Polymerization of dithieno[3,2-c:3,2-*h*][1,5]naphthyridine.

To brominate the alkylated monomer **154** derived of NT with NBS [54], the authors found that slightly elevated temperatures and a catalytic amount of acetic acid were necessary to drive the reaction and to form **203**. After purification, **203** was polymerized with 2,5-bis(trimethylstannyl) thiophene to yield **149** (PTNT, Scheme 59).

### 3.6. Metal Complex Formation

**Scheme 60:**
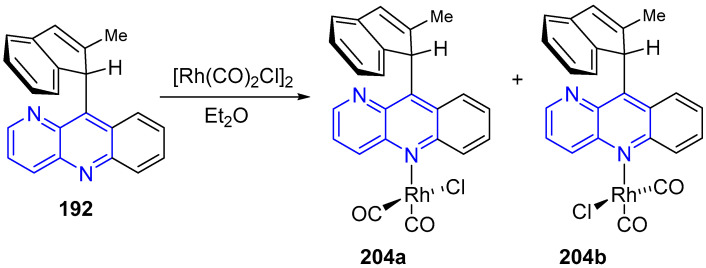
Formation of rhodium complexes.

The relative nucleophilicity of the nitrogen atoms of the 1,5-naphthyridine bicyclic system present in the benzo[*b*][1,5]naphthyridines allows these compounds to be used as ligands in the preparation of Rh, Ru, and Pd complexes. Rhodium complex bearing benzo[*b*][1,5]naphthyridine derivatives as ligands have been prepared. Thus, reaction of indenyl ligand functionalized with a benzo[*b*][1,5]naphthyridine **192** (Scheme 54, vide supra) with [Rh(CO)_2_Cl]_2_ leads to the product **204** (Scheme 60), which shows only coordination to the N5 atom [58]. Consistent with these data are isomeric structures **204a** and **204b** where the CO ligands are mutually cis; however, the structures differ from one another through restricted rotation along the Rh-N bond, due to restricted rotation of the coordinated square-planar Rh(CO)_2_Cl unit. The peri-H substituents to 5-nitrogen in the benzonaphthyridene unit are presumably responsible for this behavior (Scheme 60).

**Scheme 61:**
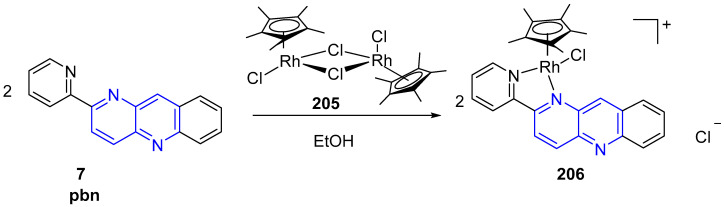
Formation of a rhodium complex from pbn.

A rhodium complex [RhCp*(pbn)Cl]Cl **206** (Scheme 61) which has a 2-(2-pyridyl)benzo[*b*] [1,5]naphthyridine **7** (pbn, Scheme 2, vide supra) and pentamethylcyclopentadienyl (Cp*) ligands, has been synthesized [59]. The complex **206** was prepared by a procedure similar to the synthesis of polypyridyl RhCp*complexes. A mixture of [RhCp*Cl_2_]_2_ **205** and two equivalents of **7** was stirred in EtOH for 1 h to give **206** (Scheme 61).

**Scheme 62:**
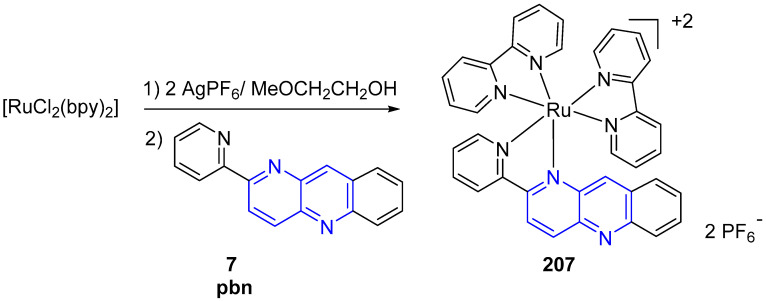
Preparation of heteroleptic pbn–ruthenium complex.

Also, ruthenium complex bearing benzo[*b*][1,5]naphthyridine derivatives as ligands have been prepared. Thus, Ru(pbn)(bpy)_2_](PF_6_)_2_ **207**, [pbn=2-(2-pyridyl)benzo[*b*][1,5]naphthyridine, bpy=2,2′-bipyridine; Scheme 62] was synthesized to study its electrochemical behavior. [RuCl_2_(bpy)_2_] was treated with two equivalents of AgPF_6_ in 2-methoxyethanol, followed by addition of one equivalent of **7** (pbn) to give [Ru(pbn)(bpy)_2_](PF_6_)_2_ **207** as a red-purple powder [11].

**Scheme 63:**
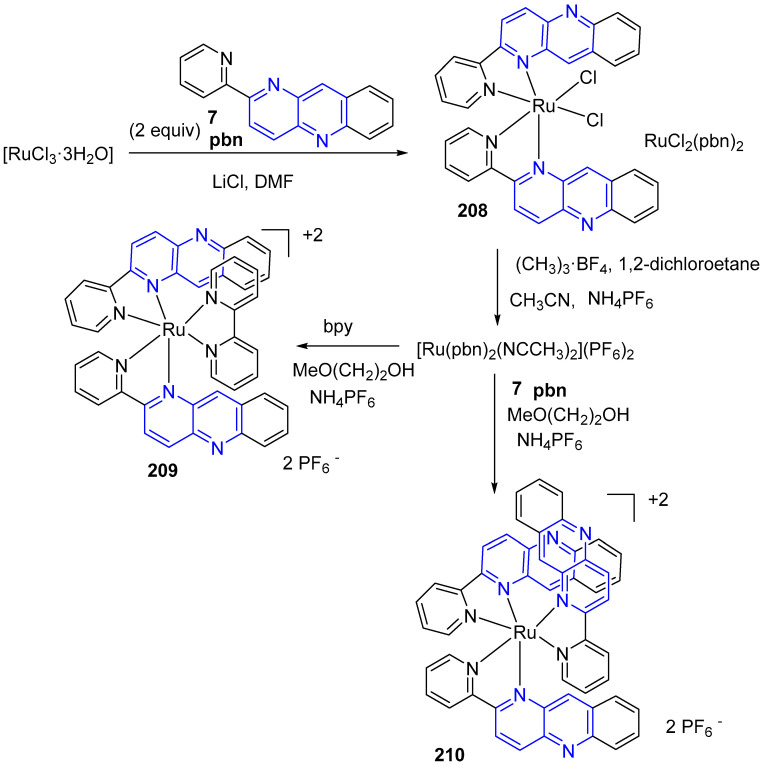
Syntheses of pbn–ruthenium complexes.

Ruthenium complexes with more than one pbn (pbn = 2-(2-pyridyl)benzo[*b*][1,5]naphthyridine) group have also been synthesized. The ruthenium complexes [Ru(bpy)(pbn)_2_](PF6)_2_ **209** (bpy = 2,2-bipyridine),and [Ru(pbn)_3_](PF6)_2_ **210** were synthesized [60] from a Ru complex with two pbn ligands **208** [RuCl_2_(pbn)_2_] (Scheme 63). Complex **208** was prepared by the reaction of RuCl_3_·3H_2_O with two equivalents of pbn in the presence of six equivalents of LiCl in DMF. Two chlorides of [RuCl_2_(pbn)_2_] **208** were removed by treatment with excess amounts of (CH_3_)_3_O·BF_4_ in 1,2-dichloroethane. After the BF_4_ anion of the crude product was exchanged with a PF_6_ anion, recrystallization of the product from CH_3_CN afforded [Ru(pbn)_2_(NCCH_3_)_2_](PF_6_)_2_. Addition of one equivalent of bpy and pbn to [Ru(pbn)_2_(NCCH_3_)_2_](PF_6_)_2_ in 2-methoxyethanol gave [Ru(bpy)(pbn)_2_](PF6)_2_ **209** and [Ru(pbn)_3_](PF_6_)_2_ **210**, respectively (Scheme 63).

**Scheme 64:**
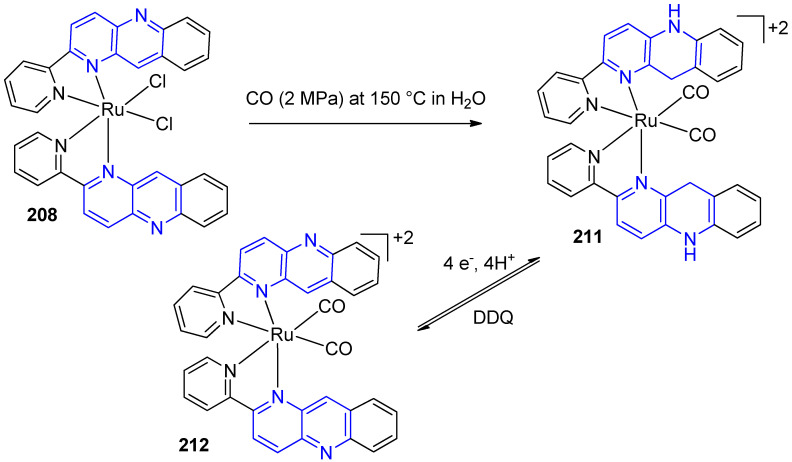
Reversible 4H^+^/4e^−^ redox reactions of the pbn–ruthenium complexes.

On the other hand, the reaction of Ru(pbn)_2_Cl_2_ **208** with CO (2 MPa) at 150 °C in H_2_O selectively produced [Ru(pbnHH)_2_(CO)_2_]^2+^ **211** accompanied by only CO_2_ evolution, indicating that [Ru(pbn)_2_(CO)_2_]^2+^ **212** initially formed in the reaction underwent 4H^+^/4e^−^ reduction under the present reaction conditions (Scheme 64) to form [Ru(pbnHH)_2_(CO)_2_]^2+^ without evolving H_2_ [61]. Furthermore, [Ru(pbnHH)_2_(CO)_2_]^2+^ **211** was quantitatively oxidized to **212** [Ru(pbn)_2_(CO)_2_]^2+^ with the treatment of two equivalents of 2,3-dichloro-5,6-dicyano-p-benzoquinone (DDQ). The reversible 4H^+^/4e^−^ redox reaction of the [Ru(pbnHH)_2_(CO)_2_]^2+^ **211**/[Ru(pbn)_2_(CO)_2_]^2+^ **212** (Scheme 64) couple without accompanying a reductive Ru-CO bond cleavage is attributable to the proton-coupled electron transfer (PCET) function of the pbn ligands.

**Scheme 65:**
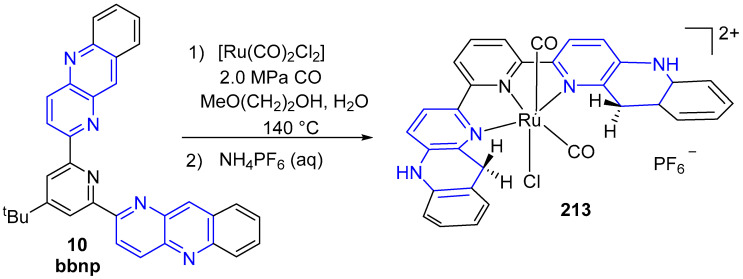
Preparation of a four-electron-reduced ruthenium(II) NADH-type complex.

A four-electron-reduced ruthenium(II) NADH-type complex, [Ru(bbnpH_4_)(CO)_2_Cl](PF_6_) **213** [Scheme 65, bbnpH_4_= 2,2′-(4-(*t*-butyl)pyridine-2,6-diyl)bis(5,10-dihydrobenzo[*b*][1,5]naphthyridine)] has been successfully synthesized by using the ligand **10** bbnp (bbnp = 2,2′-(4-(tert-butyl)pyridine-2,6-diyl)bis(benzo[*b*][1,5]naphthyridine)) and [Ru(CO)_2_Cl_2_] (Scheme 65) under moderate WGSR conditions (water-gas-shift reaction conditions) in a 1:1 ratio in a 2-methoxyethanol/water mixture (9:1 *v*/*v*) and CO pressure: 2.0 MPa, temperature: 140 °C [62].

**Scheme 66:**
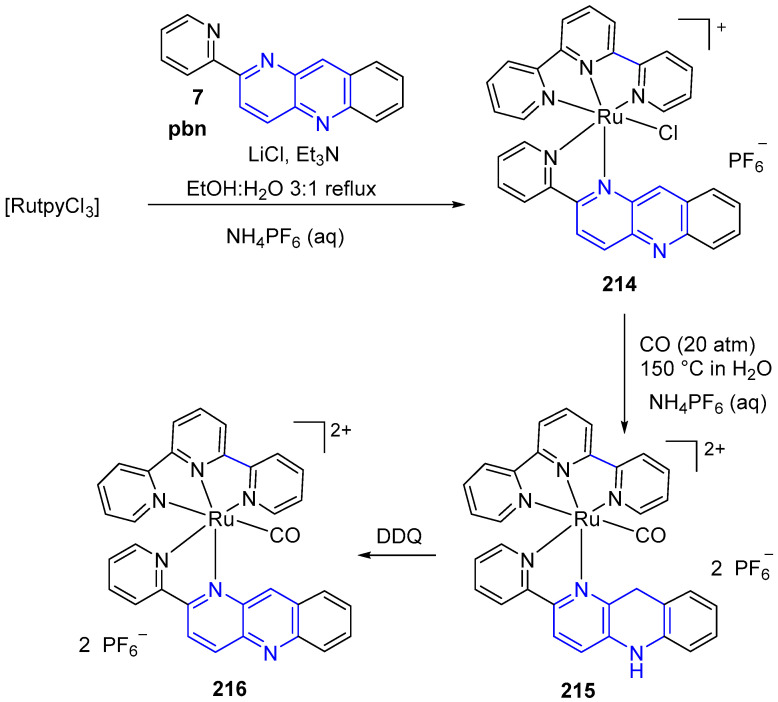
Preparation and subsequent transformations of pbn–ruthenium complexes.

In a similar way, synthesis of **216** (Scheme 66) involved three simple steps starting from Ru(tpy)Cl_3_ [63]. Conversion from **214** [Ru(tpy)(pbn)Cl]^+^ to [Ru(tpy)(pbnHH)(CO)]^2+^ **215** took place via decarboxylation process under water-gas shift reaction (WGSR) conditions (Scheme 66). [Ru(tpy)(pbnHH)(CO)]^2+^ **215** was quantitatively oxidized to [Ru(tpy)(pbn)(CO)]^2+^ **216** (Scheme 66) with the treatment of one equivalent of DDQ.

**Scheme 67:**
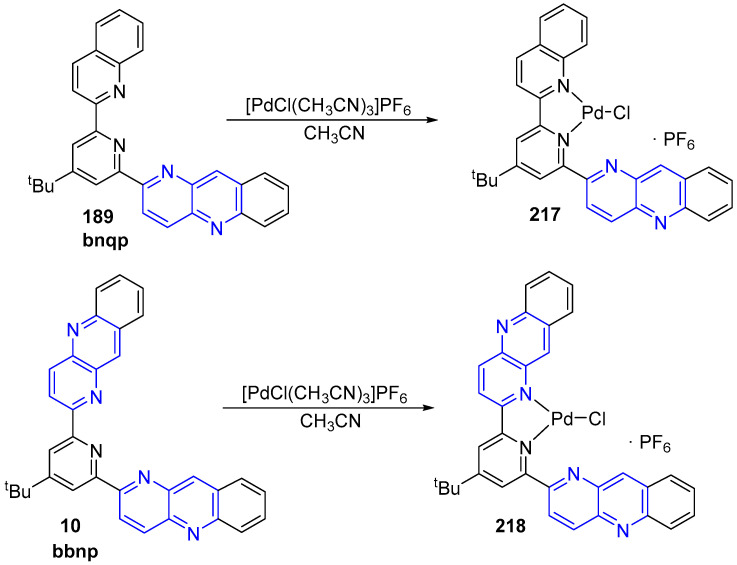
Formation of bnqp- and bbnp-palladium complexes.

Benzo[*b*][1,5]naphthyridine derivatives have been used also as ligands in palladium complexes. The palladium(**II**) complexes having the ligands, [PdCl(bnqp)](PF_6_) **217** and [PdCl(bbnp)](PF_6_) **218** (Scheme 67), were prepared by the reaction of equimolar amounts of the corresponding ligands **189** (bnqp) and **10** (bbnp) and [PdCl(CH_3_CN)_3_](PF_6_), generated in acetonitrile solution by the treatment of [PdCl_2_(CH_3_CN)_2_] with AgPF_6_ [13]. Substitution of ligand Cl by CH_3_CN was not observed under the reaction conditions, and **217** and **218** were isolated in good yields as hexafluorophosphate salts.

## 4. Biological Activity of Fused 1,5-Naphthyridines

In the last decade, complex heterocyclic systems containing 1,5-naphthyridines fragments have been synthesized, their reactions have been investigated, and the possibility of their use for the preparation of biologically active compounds have been studied extensively. For example, derivatives of annulated benzoindolo[1,5]naphthyridines attempting to improve memory were described, and these are benzo[*b*][1,5]naphthyridines which are analogs of inhibitors of neurokinin NK1-receptors [4].

**Figure 7:**
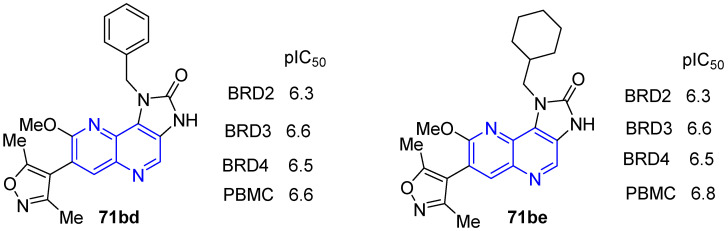
Inhibitory activity of compounds **71bd** and **71be** on BET bromodomain family.

The bromodomain and extra C-terminal (BET) domain family of bromodomains (BRDs) consists of four proteins, each containing two discrete bromodomain “reader” modules which recognize the ε-*N*-acetylation state of specific lysine residues found within histone tails and other proteins [64]. New naphthyridine analogues were synthesized and tested on BET family bromodomains [31]. Compounds 1*H*-imidazo[4,5-*c*][1,5]naphthyridin-2(3*H*)-ones **71b** (Scheme 18, vide supra) that possess an isoxazole substituent have as potent inhibitors of the BET bromodomain family with good cell activity and oral pharmacokinetic parameters. Profiling was carried out against the three BET subtypes, as well as in peripheral blood mononuclear cells (PBMCs), where inhibition of cytokine IL-6 was measured after a lipopolysaccharide (LPS) challenge. The fused indenone naphthyridines were more or less equipotent with BRD2, BRD3, BRD4 and PBMC values of pIC_50_ between 6.1 and 6.8. The best results of pIC_50_ were for compounds **71bd** and **71be** (Figure 7).

**Figure 8:**
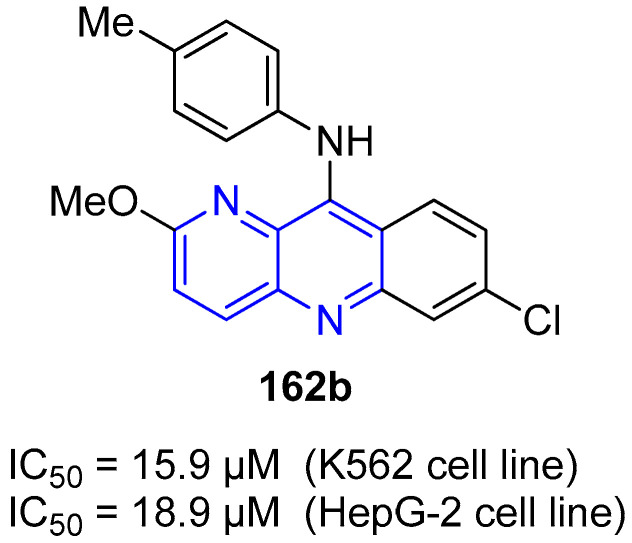
Cytotoxic activity of **162b** on K562 and HepG-2 cell lines.

Benzo[*b*][1,5]naphthyridine derivatives have been shown to be topoisomerase I (TopI) inhibitors. Compound **162b** (7-chloro-2-methoxy-*N*-(p-tolyl)benzo[*b*][1,5]naphthyridin-10-amine, Figure 8) was found to display good cytotoxicity and can bind with calf thymus DNA (ct DNA). A relaxation assay indicated that **162b** inhibits TopI activity at 100 μM [16]. Compound **162b** with methyl substitute at the para-position on the aniline ring displayed good antiproliferative activity with IC_50_ values of 15.9 and 18.9 µM against K562 and HepG-2 cells respectively in vitro. The ability of compound **162b** to interact with ct DNA indicated that nuclear enzymes involved in DNA processing such as TopI might be inhibited. These data suggest that **162b** might exert antiproliferative activity through TopI inhibition, and it may be a potential lead compound for the development of benzo[*b*][1,5]naphthyridines as TopI inhibitors.

**Figure 9:**
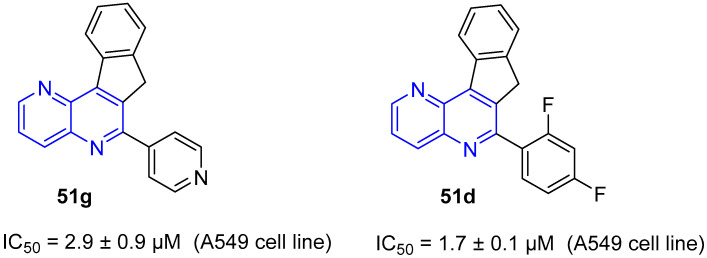
Cytotoxic activity of **51g** and **51d** on A549 cell line.

In general, 7H-indeno[2,1-*c*][1,5]naphthyridines **50** and **51** (Scheme 15, vide supra), and novel 7H-indeno[2,1-*c*][1,5]naphthyridine-7-ones **193** and 7H-indeno[2,1-*c*][1,5]naphthyridine-7-ols **194** (Scheme 45, vide supra) exhibited inhibitory effects against TopI mediated relaxation comparable to those observed for the natural inhibitor, camptothecin (CPT). All the prepared derivatives were further subjected to evaluation of their therapeutic efficacy against three different human cancer cell lines: breast (BT20), lung adenocarcinoma (A549), and ovarian carcinoma (SKOV3). These preliminary studies revealed that some of newly synthesized compounds exhibited a significant antiproliferative activity. Indeno[1,5]naphthyridine derivative **51g** (Figure 9) showed a high cytotoxic effect in vitro against A549 cell line proliferation with an IC_50_ of 2.9 ± 0.9 µM. Some of the fluorinated derivatives were the most cytotoxic, showing good enzyme inhibition and relatively low IC_50_ values. It is worth noting that indeno[1,5]naphthyridine **51d** (Figure 9) showed a very high inhibition of TopI activity and the highest cytotoxic effect, with an IC_50_ of 1.7 ± 0.1 µM, against the A549 cell line in vitro. The interesting biochemical and biological features found for these derivatives provide a promising basis for further development of biologically active fused naphthyridines [25].

The interesting biochemical and biological features found for the above derivatives provide a promising basis for further development of biologically active fused naphthyridines. Thus, tetrahydro[1,5]naphthyridine derivatives fused with heterocycles, such as chromenes **127** and chromen-2-ones **132** and the corresponding tetracyclic chromeno[4,3-*b*][1,5]naphthyridine **130** derivatives and/or chromeno[4,3-*b*][1,5]naphthyridin-6-ones **180** (Scheme 47, vide supra), showed activity as inhibitors of TopI [52]. Additionally, the cytotoxic behavior of these compounds has been studied in A549 and SKOV3 cell lines and on noncancerous lung fibroblasts cell line (MRC-5) where, on the last ones, the absence of cytotoxicity was observed.

**Figure 10:**
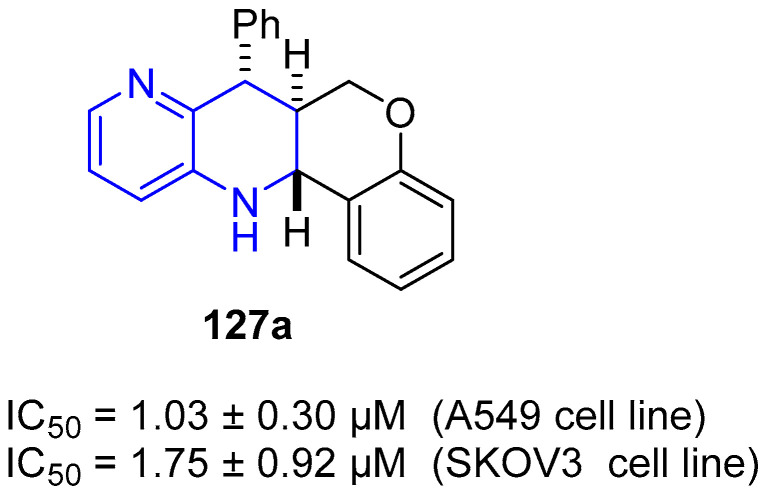
Cytotoxic activity of **127a** on A549 and SKOV3 cell lines.

7-Phenyl-6*H*-6a,7,12,12a-tetrahydrochromeno[4,3-*b*][1,5]naphthyridine **127a** (Figure 10) showed excellent cytotoxic activity with an IC_50_ value of 1.03 ± 0.30 µM against the A549 cell line and an IC_50_ value of 1.75 ± 0.20 µM against the SKOV3 cell line. The obtained results point to these compounds as good antiproliferative candidates.

**Figure 11:**
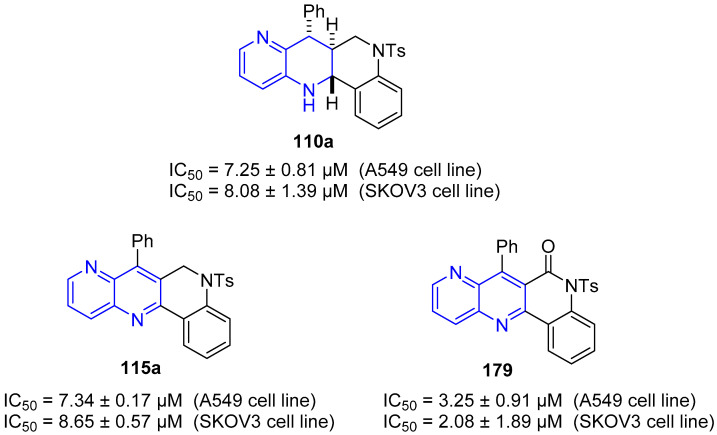
Cytotoxic activity of **110a**, **115a**, and **179** on A549 and SKOV3 cell lines.

Topoisomerase I enzymatic inhibition of hybrid quinolino[4,3-*b*][1,5]naphthyridines **110** and **115** (Scheme 47, vide supra) and quinolino[4,3-b][1,5]naphthyridin-6(5*H*)-ones **111** and **179** (Scheme 47, vide supra) was investigated [46]. The new polycyclic products show excellent-good activity as TopI inhibitors that lead to TopI induced nicking of plasmids. This is consistent with the compounds acting as TopI poisons resulting in the accumulation of trapped cleavage complexes in the DNA. The cytotoxic effect on cell lines A549, SKOV3, and on non-cancerous MRC-5 was also screened. Tetrahydroquinolino[4,3-*b*][1,5]naphthyridin-6(5H)-one **179** (Figure 11) resulted the most cytotoxic compound with IC_50_ values of 3.25 ± 0.91 μM and 2.08 ± 1.89 μM against the A549 cell line and the SKOV3 cell line, respectively. Moreover, 5-tosylhexahydroquinolino[4,3-*b*][1,5]naphthyridine **110a** and 5-tosyldihydroquinolino[4,3-*b*][1,5]naphthyridine **115a** (R^1^ = H) demonstrated to be cytotoxic with IC_50_ values of 7.25 ± 0.81 μM and 7.34 ± 0.17 μM against the A549 cell line, respectively, and with IC_50_ values of 8.08 ± 1.39 μM and 8.65 ± 0.57 μM against the SKOV3 cell line, respectively. None of the compounds had cytotoxic effects against non-malignant pulmonary fibroblasts (MRC-5).

**Figure 12:**
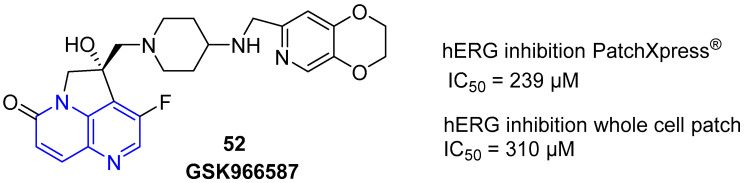
Inhibition of **52** on hERG.

Novel 4,5-dihydro-6λ^4^pyrrolo[3,2,1-*ij*][1,5]naphthyridine hydroxy-derivatives including **52** GSK966587 (Figure 12) have been discovered as inhibitors of bacterial type IIA topoisomerases. The compounds showed good potency against Gram-positive and Gram-negative pathogens [28]. The hERG inhibition of the compounds described was generally related to their lipophilicity and basicity and several compounds within this series were identified with hERG inhibition (Figure 12).

**Figure 13:**
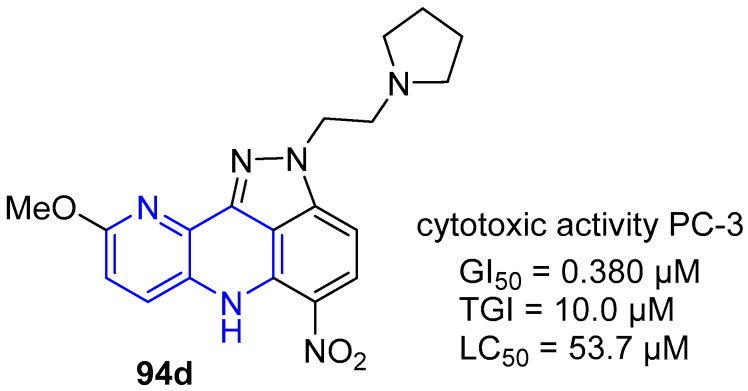
Cytotoxic activity of **94d** on PC-3 prostate cancer cells.

The antitumor activity of fused 1,5-naphthyridines derivatives is closely connected with the intercalation characteristic of many planar ionisable systems. 2-[(Alkylamino)alkyl]-9-methoxy -5-nitro-2,6-dihydroindazolo[4,3-*bc*][1,5]naphthyridines **94** (Scheme 24, vide supra) represent a new class of aza-acridine derivatives which have noticeable antitumor properties [15]. With enhanced DNA affinity and similar in vitro cytotoxic activity in respect to reference compound (pyrazinamide) PZA, but specially, with a capacity to induce oligonucleosomal DNA fragmentation and apoptotic cell death, not present in PZA. In particular the 9-methoxy-5-nitro-2-[2-(tetrahydro-1*H*-1-pyrrolyl) ethyl]-2,6-dihydroindazolo[4,3-*bc*][1,5]naphthyridine **94d**, which possesses the most relevant biological characteristics in the series, can be regarded as a new lead in the field of potential anticancer derivatives (Figure 13). Thus, the ability of this compound to early induce oligonucleosomal DNA fragmentation and apoptotic cell death of the hormone-refractory PC-3 prostate cancer cells may be particularly relevant to overcoming drug resistance or sensitize tumor cells to the effects of other antineoplastic agents.

**Figure 14:**
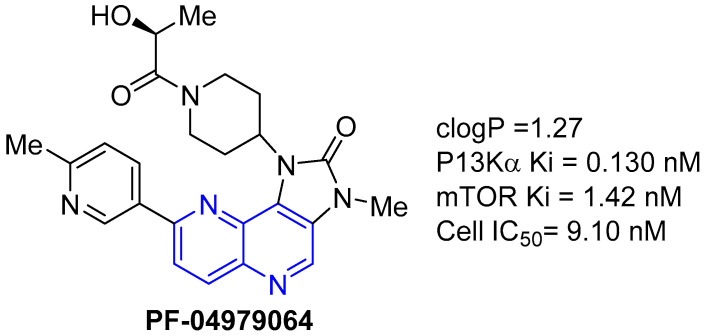
PI3K/mTOR dual kinase inhibition of PF-04979064.

To search for a structurally differentiated backup candidate to PF-04691502 (Figure 14), which is currently in phase I/II clinical trials for treating solid tumors, a lead optimization effort was carried out with a tricyclic imidazo[1,5]naphthyridine series. Integration of a structure-based drug design and physical properties-based optimization (Scheme 18, vide supra) yielded PF-04979064, a potent and selective PI3K/mTOR dual kinase inhibitor [30].

The manuscript discusses the lead optimization for the tricyclic series, which both improved the in vitro potency and addresses a number of absortion, distribution, metabolism, excretion and toxicity (ADMET) properties including high metabolic clearance mediated by both P450 and aldehyde oxidase (AO), poor permeability, and poor solubility. An empirical scaling tool was developed to predict human clearance from in vitro human liver S9 assay data for tricyclic derivatives that were AO substrates.

**Figure 15:**
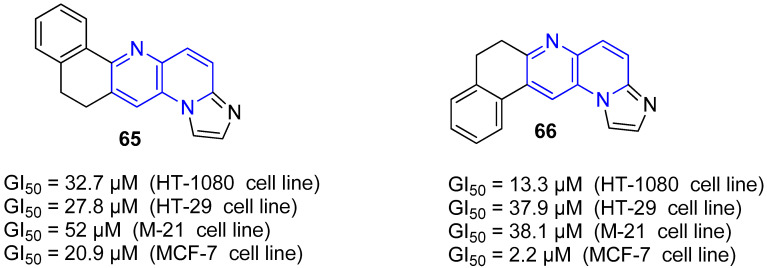
Cytotoxic activity of **65** and **66** on HT-1080, HT-29, M-21, and MCF-7 cell lines.

11,12-Dihydroimidazo[1,2-a]naphtho[1,2-*g*][1,5]naphthyridine **65** and 7,8-dihydroimidazo [1,2-*a*]naphtho[2,1-*g*][1,5]naphthyridine **66** (Scheme 17, vide supra) exhibited in vitro activity comparable to anticancer agent such as amsacrine [29]. Their mechanism of cytotoxicity action was unrelated to poisoning or inhibiting abilities against TopI. On the contrary, must be highlighted a direct intercalation of the drug into DNA by electrophoresis on agarose gel. The tumor cell growth inhibition was assessed on HT-1080 (fibrosarcoma), HT-29 (colon carcinoma), M-21 (skin melanoma), and MCF-7 (breast carcinoma) cells. Compounds **65** and **66** exhibited good values of GI_50_ ranging from 2.2 to 52 μM (Figure 15). Compound **66** seems to exhibit some specificity towards breast-derived cells such as MCF-7 where it showed a GI_50_ at least fourfold higher than in any other tumor cell lines (Figure 15).

**Figure 16:**
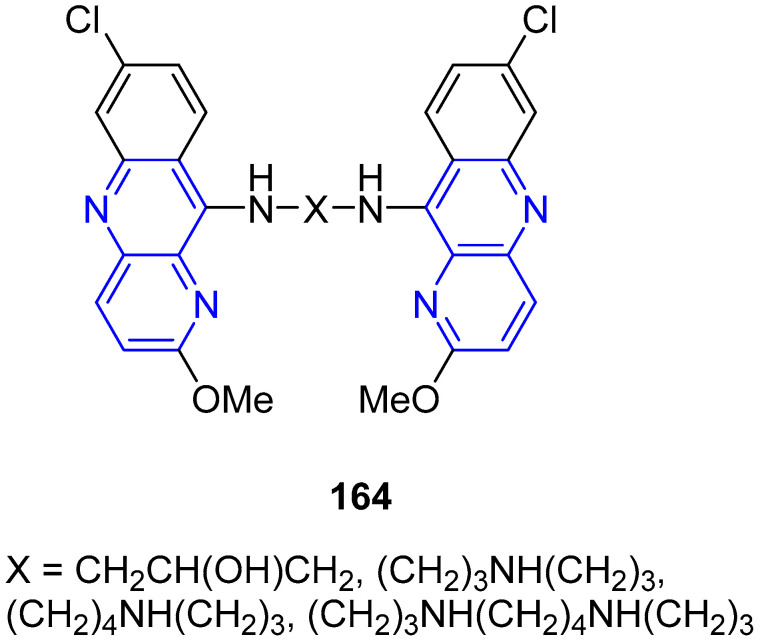
Dimeric benzo[*b*][1,5]naphthyridines **164** as bis-acridine analogs.

A library of dimeric benzo[*b*][1,5]naphthyridines **164** (Figure 16) was synthesized to explore the effect of structurally diverse linkers on PrP^Se^ replication in scrapie-infected neuroblastoma cells [4]. The data suggest that bis-acridine analogs may provide a potent alternative to the acridine-based compound quinacrine which is currently under clinical evaluation for the treatment of prion disease.

**Figure 17:**
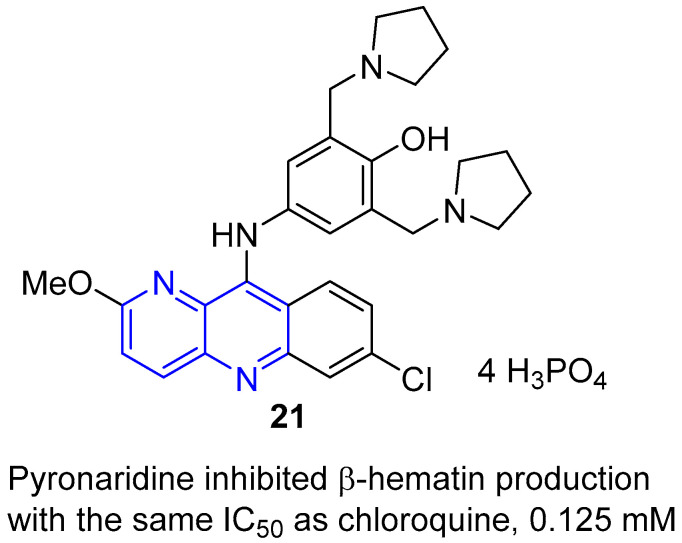
β-Hematin inhibition of **21**.

Pyronaridine, 4-((7-chloro-2-methoxybenzo[*b*][1,5]naphthyridin-10-yl)amino)-2,6-bis (pyrrolidin-1-ylmethyl)phenol **21** (Figure 17), a new Mannich base, schizontocide, originally developed in China and structurally related to the aminoacridine drug quinacrine, is currently undergoing clinical testing. Pyronaridine targets hematin, as demonstrated by its ability to inhibit in vitro β-hematin formation (at a concentration equal to that of chloroquine), to form a complex with hematin with a stoichiometry of 1:2, to enhance hematin-induced red blood cell lysis (but at 1/100 of the chloroquine concentration), and to inhibit glutathione dependent degradation of hematin. Pyronaridine exerted this mechanism of action in situ, based on growth studies of *Plasmodium falciparum* K1 in culture [65].

**Figure 18:**
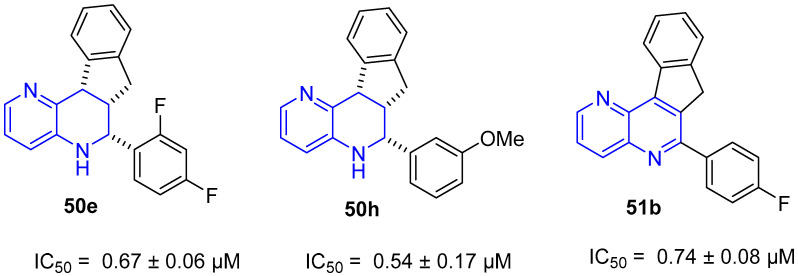
Antileishmanial activity of **50e**, **50h**, and **51b**.

The inhibition of leishmania (LTopIB) and human TopIB (HTopIB) of tetrahydroindeno[1,5]naphthyridines **50** and indeno[1,5]naphthyridines **51** (Scheme 15, vide supra) were studied and their antileishmanial activity on promastigotes and amastigote-infected splenocytes of *Leishmania infantum* were evaluated [25]. Some of the prepared heterocycles showed selective inhibition of LtopIB, while no inhibition of hTopIB was observed at evaluated conditions. In addition, the cytotoxic effects of newly synthesized compounds were assessed on host murine splenocytes in order to calculate the corresponding selective indexes (SI). Tetrahydro indeno[1,5]naphthyridines **50e** and **50h** (Figure 18) showed good antileishmanial activity (IC_50_ values of 0.67 ± 0.06 and 0.54 ± 0.17 µM) with similar activity than the standard drug amphotericin B (0.32 ± 0.05 µM) and even tetrahydro indeno[1,5]naphthyridine **50h** showed higher (SI) towards *L. Infantum* amastigotes. Likewise, in the family of indeno[1,5]naphthyridines **51**, compound **51b** (Figure 18) showed good antileishmanial activity (IC_50_ value 0.74 ± 0.08 µM) and higher selective index towards *L. Infantum* amastigotes than amphotericin.

**Figure 19:**
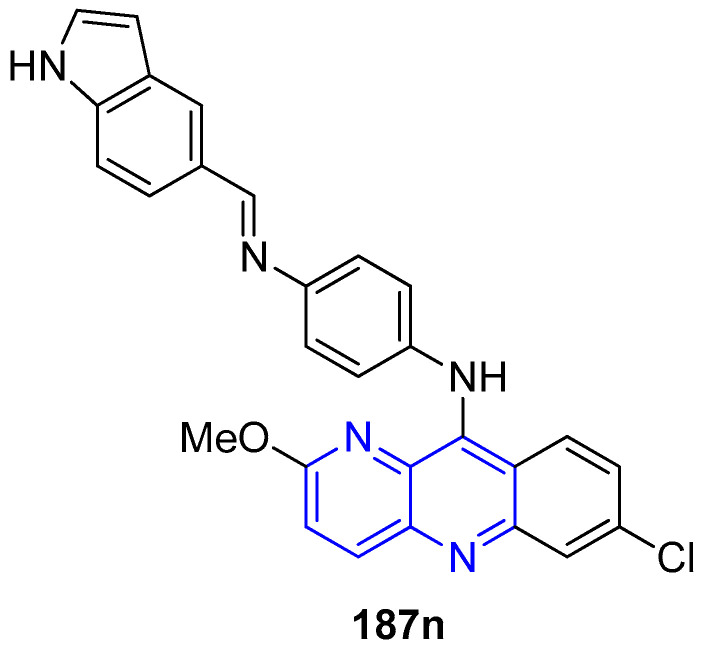
Activity of **187n** against *Nippostrongylus brazilliensis*.

The anti-intestinal nematode activities against *Nippostrongylus brazilliensis* of a series of benzonaphthyridine derivatives bearing the C=N linkage moiety **187** (Scheme 51, vide supra) were evaluated in vivo by an oral route in male rats [57]. Some of compounds showed significant anti-intestinal nematode activity in a two-day in vivo test in rats. Among these compounds, at concentrations of 10 mg/kg of rat, the compound 7-chloro-2-methoxy- 10-(4-(4′-(1H-indol-5′-yl) methylene)aminophenyl)-amino-benzo[*b*][1,5]naphthyridine **187n** (Figure 19) produced the highest activity against *Nippostrongylus brazilliensis*, with 80.3% deparasitization. These compounds may find usefulness in the discovery and development of new anti-intestinal drugs.

**Figure 20:**
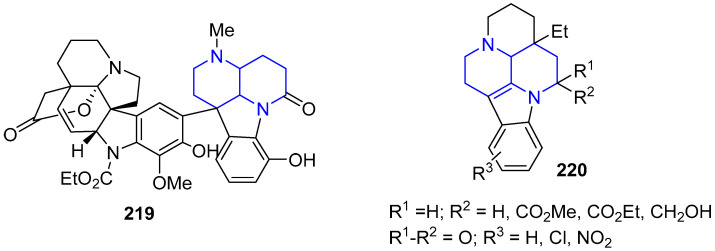
Fused 1,5-naphthyridines with hypotensive and pain-relieving activities.

The dimeric indole alkaloid cimiciduphytine **219** (Figure 20) containing the [1,5]naphthyridine fragment and derivatives of eburnane alkaloids **220** (Figure 20) exhibiting hypotensive and pain-relieving activities have been isolated from naturally occurring sources [4]. These compounds are suitable for the treatment of cerebral circulation disturbance.

## 5. Other Applications of Fused 1,5-Naphthyridines

**Scheme 68:**
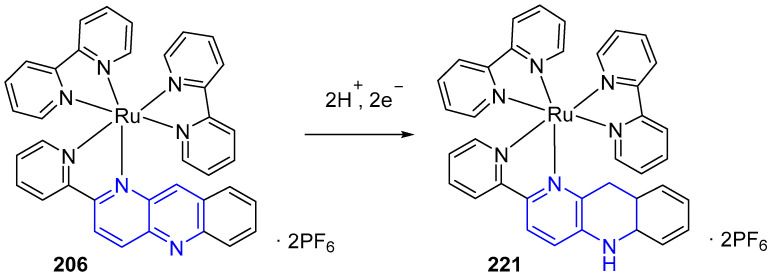
Formation of the NADH-like species from bpn–ruthenium(II) complex.

In biological reactions, on the other hand, the NAD+/NADH redox couple, in which the oxidized form (NAD+) with a pyridinium structure is reversibly converted into the reduced form (NADH) with two electrons and one proton, plays a key role in reversible hydride transfer reactions. Transition-metal complexes operating in the same way as the NAD+/NADH system have been reported. Many of these systems present in their structure model ligands that include benzo[*b*][1,5]naphthyridin-2-yl groups. The electrochemical reduction of **206** (Scheme 68) under aqueous acidic conditions induces formation of the hydrogenated product [Ru(pbnH_2_)(bpy)_2_](PF_6_)_2_] **221** [11]. The electrochemical reduction of acetone to generate 2-propanol by using complex **206** as a precatalyst with two electrons and H_2_O as a proton source was described. The key points of this catalytic system are “hydride” generation and transfer, similar to the function of the NAD+/NADH redox couple. Clear evidence of the photochemical and radiolytic formation of **221** with H^+^ have been reported in Reference [66]. The mechanistic pathways of formation of the NADH-like [Ru(bpy)_2_(pbnHH)]^2+^ species from [Ru(bpy)_2_(pbn)]^2+^ were studied in an aqueous medium [67] and D_2_O [68] showing is controlled by the pH of the solution.

On the other hand, on study demonstrated that the introduction of a methyl group of the pyridine moiety in the pbn ligands played a key role in controlling photo-induced NAD+/NADH type hydrogenation reactions by twisting the ligand at a certain level compared with the case of the non-substituted pbn ligand [69].

**Figure 21:**
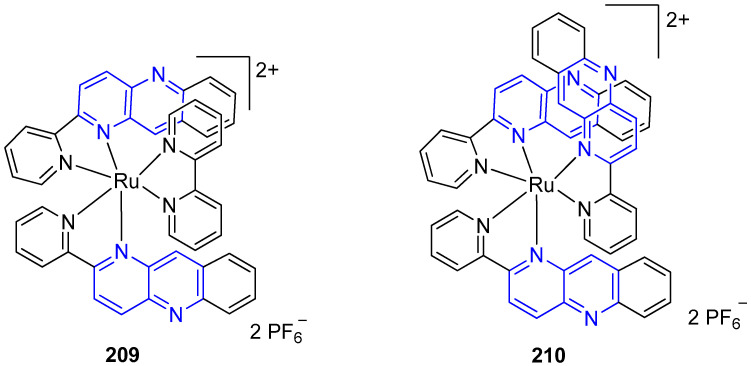
Ruthenium complexes containing fused 1,5-naphthyridines.

The use of the multielectron redox reactions of mononuclear metal complexes under visible light irradiation is a fascinating approach to harvesting solar energy. The photoinduced four- and six-electron reduction of [Ru(bpy)(pbn)_2_](PF_6_)_2_ **209** and [Ru(pbn)_3_](PF_6_)_2_ **210** (Figure 21), with two and three pbn ligands, respectively, under irradiation with visible light (l > 420 nm) conducted in dry CH_3_CN/TEA (4:1, *v*/*v*) resulted in only one-electron reduction of these complexes to give (**209**)^+^ and (**210**)^+^. The high-efficiency storage of photoinduced two-, four-, and six-electron reducing equivalents in **207** (Scheme 68, vide supra), **209** and **210**, respectively, using the NAD+ analogous pbn ligands may provide a new pathway for multiple electron and proton transfer to reaction sites under illumination with visible light [60].

The design and synthesis of new catalysts having a capability for photo- and electro-chemical reduction of carbon dioxide (CO_2_) to produce CO, HCOOH, alcohols, etc., have been pursued to alleviate the global crisis caused by depletion of fossil fuels and the rising atmospheric concentration of CO_2_. Among the various catalysts, ruthenium complexes may be viable candidates to realize such an innovative function, since they are widely used as catalysts in photo- and electrochemical reduction of CO_2_ as well as a variety of organic syntheses. The development of a renewable hydride donor is a key process to construct a catalytic system that has the ability to catalyze multi-electron reduction of CO_2_. Reduction of CO_2_ using the renewable hydride donor embarks on a new stage of the construction of a sustainable society [70].

**Scheme 69:**
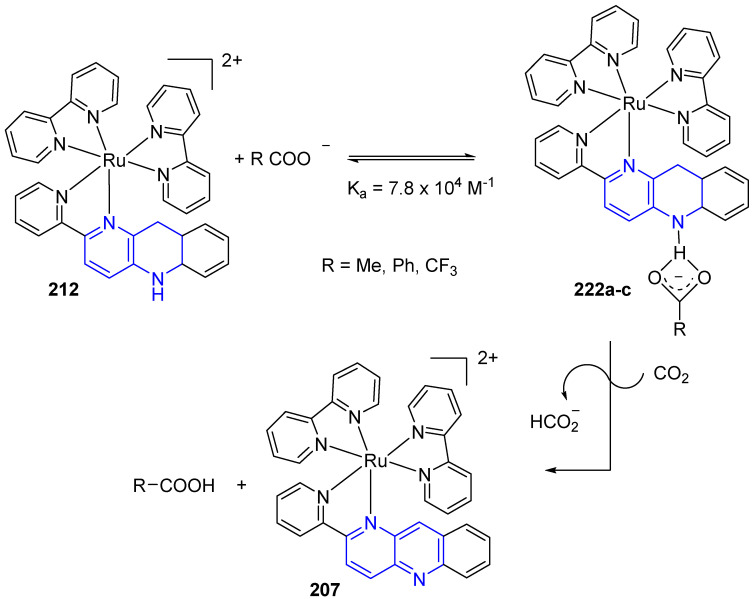
Photo- and electrochemical reduction of CO_2_ with bpn–ruthenium complexes.

An addition of a base to Ru-pbnHH greatly enhanced the hydride donor ability, since [Ru(bpy)_2_(pbnHH)]^2+^ **212** reacted with CO_2_ in the presence of PhCOO^_^ to give HCOO^_^ with regeneration of **207** [Ru(bpy)_2_(pbn)]^2+^ [71]. [Ru(bpy)_2_(pbnHH)]^2+^ **212** was mixed with PhCOO^−^ at −40 °C under CO_2_ (Scheme 69, R = Ph). The ESI-MS and ^1^H NMR spectra of the reaction mixture at that temperature displayed 1:1 adduct formation between PhCOO^−^ and [Ru(bpy)_2_(pbnHH)]^2+^ **222a** (Scheme 69, R = Ph). The association constant (K_a_) was 7.8 × 10^4^ M^−1^.

**Scheme 70:**
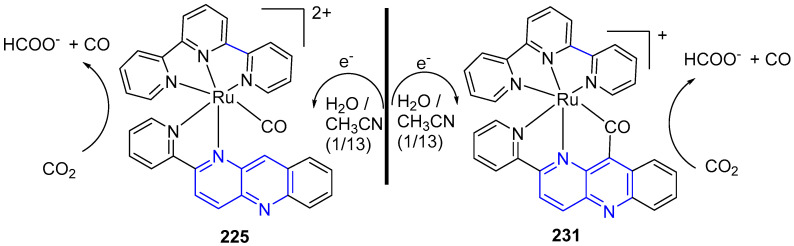
Electrochemical reduction of CO_2_ using bpn–ruthenium–NAD-type complexes.

A drastic difference in the organic hydride transfer reaction converting CO_2_ to HCO_2_^−^ using the ruthenium complex containing the NADH model ligand **212** (Scheme 69) was observed by changing the base to either acetate anion (MeCOO^−^, Scheme 69, R = Me) or trifluoroacetate anion (CF_3_COO^−^, (Scheme 70, R = CF_3_) [72]. The study demonstrated that the choice of the base plays a key role in the CO_2_ reduction system utilizing **212** through the association of the base to the NH moiety of the NADH model ligand pbnHH (Scheme 69); the difference in basicity between MeCOO^−^ and CF_3_COO^−^ led to notable accelerating and decelerating effects on the rate of the organic hydride transfer reaction as compared to PhCOO^−^.

In this context, the electrochemical reduction of CO_2_ using the catalytic performance of Ru-NAD-type complexes [Ru(tpy)(pbn)(CO)]^2+^ **216**; tpy = 2,2;6,2-terpyridine; pbn = 2-(pyridin-2-yl)benzo[*b*][1,5]naphthyridine and the Ru-CO-bridged metallacycle **223** was investigated in H_2_O/CH_3_CN at room temperature [73]. A controlled-potential electrolysis of **216** and **223** afforded formate (HCOO^−^) as the main product, under concomitant formation of minor amounts of CO and H_2_ (Scheme 70).

**Figure 22:**
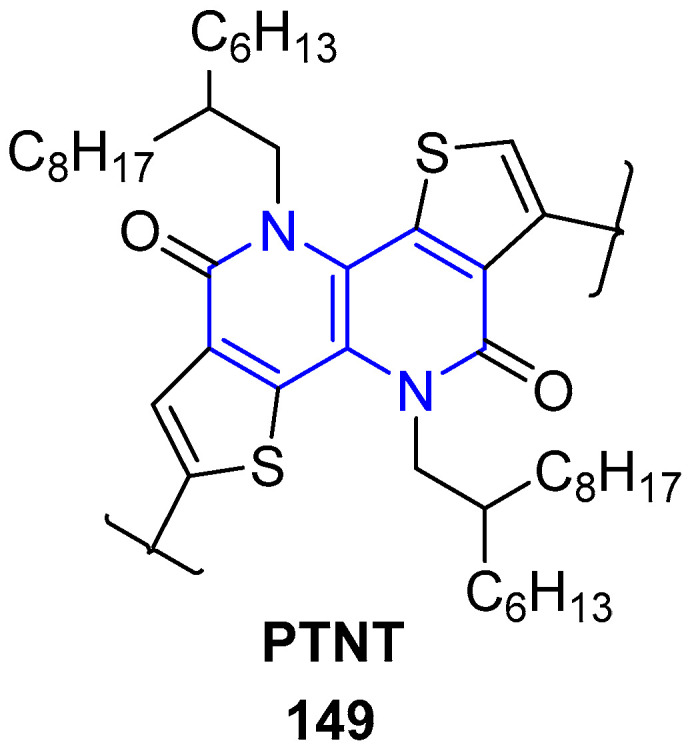
Fused 1,5-naphthyridine with OLED properties.

PTNT (poly[thiophene-2,5-diyl-*alt*-5,10-bis((2-hexyldecyl)oxy)dithieno[3,2-c:3′,2′-*h*][1,5]- naphthyridine-2,7-diyl]) **149** consists of a tetracyclic fused lactim ring (see Figure 22) that offers many favorable properties, such as improved solid-state packing, high charge carrier mobility, and moderate photoluminescence quantum yields (PLQYs), required for fast optoelectronics. Photophysical properties of PTNT were explored in solution and thin film. Thus, a new strategy to improve the speed of organic light-emitting diodes (OLEDs) using a new class of luminescent polymer with high charge carrier mobilities has been demonstrated [74]. Aqueous nanoparticle dispersions were prepared from PTNT and fullerene blend utilizing chloroform as well as a non-chlorinated and environmentally benign solvent, o-xylene, as the miniemulsion dispersed phase solvent. The nanoparticles (NPs) in the solid-state film were found to coalesce and offered a smooth surface topography upon thermal annealing [75]. Recently, PTNT-conjugated polymer was used as the donor polymer. The preparation of environmentally friendlier polymer solar cell devices [76,77].

**Figure 23:**
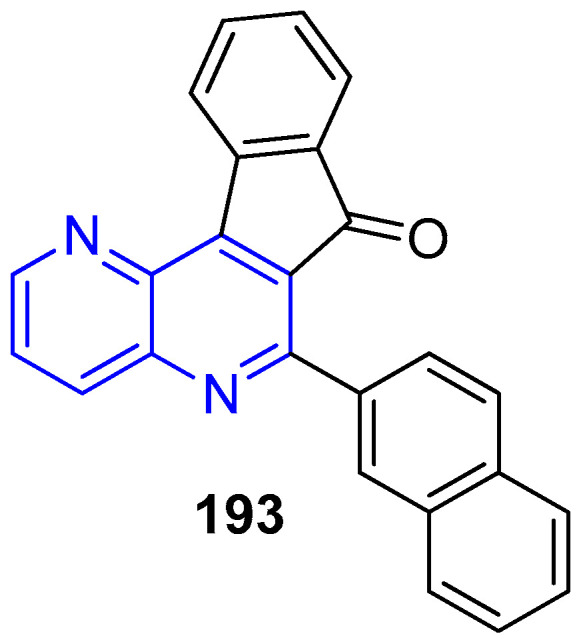
Fused 1,5-naphthyridine as optical DNA biosensor.

The indeno[2,1-*c*][1,5]naphthyridine-7-one **193** (Figure 23), which has a 2-naphthyl group, has been used as an optical DNA biosensor to unravel the inhibitory mechanism of human topoisomerase I activity by blocking enzyme—DNA dissociation [78]. This represents the first characterized example of a small molecule drug that inhibits a post-ligation step of catalysis.

**Scheme 71:**
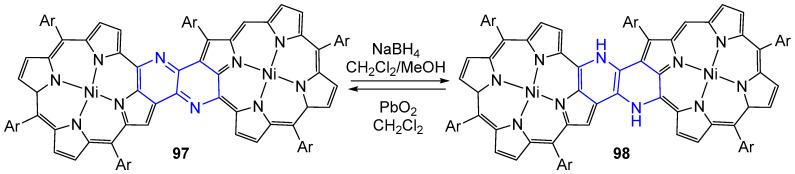
Redox system of 1,5-naphthyridine-fused porphyrin dimers.

The dimmer **98** is quite electron-rich owing to the presence of a 1,2-diaminoethene bridge and is oxidized back to **97** (Scheme 71) within several hours in solution under ambient conditions. Furthermore, **97** possesses a redox-active 1,4-diazabutadiene linkage that is interconvertible with its reduced 1,2-diaminoethene linkage upon treatment of **97** with NaBH_4_ or PbO_2_. The dimmer **97** exhibits an intense NIR absorption and narrow HOMO-LUMO gap with a remarkably low reduction potential mainly due to the effective bonding interactions in the LUMO through the 1,4-diazabutadiene linkage. In contrast, the reduced dimmer **98** has high HOMO energy and shows a relative large HOMO-LUMO gap compared to that of **97** [39].

**Figure 24:**
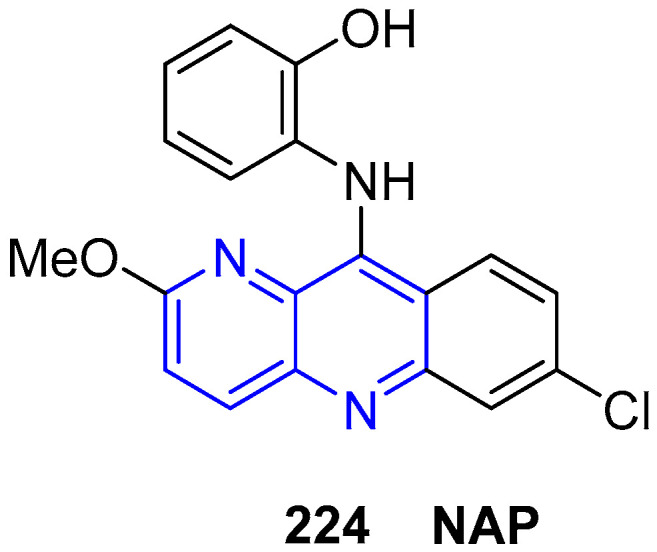
Fused 1,5-naphthyridine as colorimetric sensor for detection of Cu^2+^ ions.

A highly selective and sensitive acridine-base colorimetric sensor 2-((7-chloro-2-methoxybenzo[*b*]1,5]naphthyridine-10-yl)amino)phenol **224** (NAP, Figure 24) was developed for detection of Cu^2+^ ions both in aqueous solution and on test papers. Sensor NAP responses to Cu^2+^ ions by changing its color from yellow to pink, which could be easily observed by the naked eyes [79].

## 6. Conclusions

The contributions published in the last 18 years related to the synthesis and reactivity of the fused 1,5-naphthyridine derivatives were analyzed. According to the data presented, these types of fused heterocycles have attracted great interest, not only for synthetic but also for medicinal chemists.

Some fused 1,5-naphthyridines presented in this review show important biological activity as enzymatic inhibitors with antiproliferative, antiparasitic and antibacterial capacities. Likewise, some polycyclic 1,5-naphthyridines has also been reported as a biological sensor for many techniques. Moreover, some fused heterocycles show applications as light-emitting compounds, CO_2_ reductors, and hydride donors.

In this sense, this revision could be useful to synthetic and medicinal chemists because of the information related to the synthesis and biological activity of fused 1,5-naphthyridines and, on the other hand, by taking advantage of the electronic and optical properties of the described heterocycles, progress could be made in the development of new technologies.
